# Exploring Lymph Node Stroma Ageing: Immune Implications and Future Directions

**DOI:** 10.1111/acel.70000

**Published:** 2025-02-15

**Authors:** Yu Yang Ng, Andy Tay

**Affiliations:** ^1^ Department of Biomedical Engineering National University of Singapore Singapore City Singapore; ^2^ Institute for Health Innovation & Technology National University of Singapore Singapore City Singapore; ^3^ Tissue Engineering Programme National University of Singapore Singapore City Singapore

**Keywords:** ageing, immunity, infections, lymph node, stromal cells

## Abstract

Ageing is an inevitable biological process that impacts the immune system, leading to immunosenescence and inflammaging, which contribute to increased susceptibility to infections, autoimmune diseases and cancers in individuals over the age of 65. This review focuses on the ageing of lymph node stromal cells (LNSCs), which are crucial for maintaining lymph node (LN) structure and function. Age‐related changes in LNs, such as fibrosis and lipomatosis, disrupt the LN architecture and reduce immune cell recruitment and function, impairing immune responses to infections and vaccinations. The review discusses the structural and functional decline of various LNSC subsets, including fibroblastic reticular cells (FRCs), lymphatic endothelial cells (LECs) and blood endothelial cells (BECs), highlighting their roles in immune cell activation and homeostasis. Potential strategies to restore aged LNSC function, such as enhancing LNSC activation during vaccination and using senotherapeutics, are explored. Outstanding questions regarding the mechanisms of LNSC ageing and how ageing of the LN stroma might impact autoimmune disorders are also addressed. This review aims to stimulate further research into the characterisation of aged LNSCs and the development of therapeutic interventions to improve immune function in the older adults.

## Graphical abstract

This review examines how ageing affects lymph node stromal cells (LNSCs), leading to structural changes like fibrosis and lipomatosis that impair LN architecture and immune function. It also discusses potential interventions to improve aged LNSC function, including senotherapeutics and vaccination strategies, while posing critical questions about the mechanisms driving LNSC ageing and the possible link between aged LN stroma and autoimmune disorders.

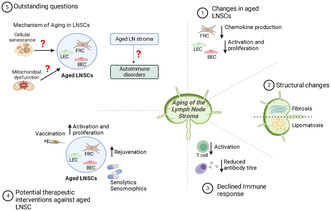

## 1 Introduction

Ageing is a natural and unavoidable process in living organisms. As individuals age, their immune systems typically experience a decline in function, referred to as immunosenescence (Fulop et al. 2015; Yu and Zheng 2019; Feehan, Tripodi, and Apostolopoulos 2021). This weakening of the immune system, combined with inflammaging (a state of chronic inflammation), contributes to a higher occurrence of infections, autoimmune diseases and cancers in people aged 65 years and above (Morrisette‐Thomas et al. 2014). Moreover, older adults often exhibit diminished responses to vaccinations compared to younger individuals (Osterholm et al. 2012; Bell and Kutzler 2022). The ageing process affects various aspects of the immune system, including both innate (Solana et al. 2012; Hazeldine and Lord 2013; Montgomery and Shaw 2015) and adaptive (Goronzy et al. 2015; Yanes et al. 2017) components, the microenvironment of lymphoid organs (Masters et al. 2017; Thompson et al. 2017; Lancaster 2023; Sonar, Watanabe, and Nikolich 2023) where immune cells develop and reside, and the balance of soluble chemokines and cytokines that play a crucial role in immune system functioning and homeostasis. While the changes in the primary lymphoid organs like the bone marrow (Pritz, Weinberger, and Grubeck‐Loebenstein 2014; Pangrazzi et al. 2017; Naismith and Pangrazzi 2019; Ho and Méndez‐Ferrer 2020) and thymus (Hamazaki, Sekai, and Minato 2016; Liang et al. 2022; Kousa et al. 2024) with ageing are well‐documented, information regarding the ageing of secondary lymphoid organs such as lymph nodes (LN) remains incomplete and requires further exploration and discussion.

Studies have indicated that as individuals age, there is a reduction in the size of the LN, accompanied by degenerative changes such as the development of fibrosis and lipomatosis (Luscieti et al. 1980; Hadamitzky et al. 2010; Ahmadi, McCall, and Stringer 2013; Jin et al. 2022; Pan, Suami, and Taylor 2008; Gödde et al. 2023; Erofeeva and Mnikhovich 2018, 2020). There is also evidence of changes in the structure of the LN endothelium, leading to decrease in immune cell recruitment (Jin et al. 2022; Bekkhus et al. 2023). As a result, the immune cell number present in the LN diminishes. Furthermore, during ageing, the number and size (area) of germinal centres (GC) is reduced by approximately 30%–50% (Luscieti et al. 1980; Hadamitzky et al. 2010; Ahmadi, McCall, and Stringer 2013; Pan, Suami, and Taylor 2008). This deficit results in reduced humoral immunity, leading to impaired antibody production and an increased susceptibility to infections in individuals over the age of 65 (Lee and Linterman 2022; Silva‐Cayetano et al. 2023). Consequently, it can be speculated that the disorganisation of the LN structure play a significant role in the ageing of the immune system.

The architecture of the LN is created and supported by LN stroma cells (LNSCs), comprising heterogeneous populations of mesenchymal cells and endothelial cells (Krishnamurty and Turley 2020; Grasso et al. 2021; Zou, Wiechers, and Huehn 2021; Chang and Turley 2015). LNSCs organise the LN into distinct compartments to support immune cell retention, activation, proliferation and differentiation in homeostatic conditions and in response to antigenic stimulation (Krishnamurty and Turley 2020; Grasso et al. 2021; Zou, Wiechers, and Huehn 2021; Chang and Turley 2015). To support and maintain the distinct yet diverse immune cell niches within the LN, LNSCs secrete various growth factors and chemokines to ensure immune cells are correctly localised to their unique niches and receive appropriate survival signals (Krishnamurty and Turley 2020; Grasso et al. 2021; Zou, Wiechers, and Huehn 2021; Chang and Turley 2015). Thus, LNSC plays an important role in ensuring immune cell homeostasis, activation of immune responses during infection, and accordingly, any age‐associated changes to LNSCs may significantly hamper the overall function of the LN as a hub for immunosurveillance. As a matter of fact, studies performed in the recent years have begun to shed light how age‐associated changes to LNSCs impairs the generation of protective immunity against infection and after vaccination (Silva‐Cayetano et al. 2023; Becklund et al. 2016; Thompson et al. 2019; Kwok et al. 2022; Richner et al. 2015; Masters et al. 2019; Denton et al. 2022). The observation that the older adults are not able to generate effective long‐term protective immunity after vaccination highlights the necessity to comprehend how underlying age‐associated changes to LNSC precipitates into impaired immune responses, and further research may potentiate development of therapeutic strategies that could enhance immune responses by targeting the aged LNSCs.

This review will begin by describing how the LN stroma is affected at the cellular and structural levels during ageing and how these age‐associated changes impact immune responses during infection or vaccination. It will also discuss current efforts aimed at improving immune responses in individuals over the age of 65, and how these strategies may be applicable to the aged LN stroma. Finally, outstanding questions in the field will be briefly highlighted. It is hoped that this review will prompt further efforts to better characterise the stromal population in aged LNs to enhance our understanding of their contribution to age‐associated declines in immunity.

## 2 Overview of the Stromal Cell Components of the LN

LNs are organised into compartments, where different areas are dedicated to the support and function of various immune cell type. Specialised lymph and blood vessels provide access and transport for lymph and blood. Within this framework, stromal cells create a mesh network within the LN, facilitating the movement of adaptive and innate immune cells throughout the LN. Various subpopulations of stromal cells have been identified in LNs (Krishnamurty and Turley 2020; Grasso et al. 2021; Zou, Wiechers, and Huehn 2021; Chang and Turley 2015), differentiated by their surface markers, such as podoplanin (PDPN; gp38) and CD31 (PECAM). These subsets include fibroblastic reticular cells (FRCs; PDPN+CD31−), lymphatic endothelial cells (LECs; PDPN+CD31+), blood endothelial cells (BECs; PDPN−CD31+) and a double‐negative population (DN; PDPN−CD31−). Currently, stromal cells that may be present within this DN cell population includes pericytes (gene signature: *Itga7+Pdgfrb+*) and smooth muscle cells (SMC) (gene signature: *Myh11+*), in which they are identified either by single cell RNA (scRNA) sequencing and/or immunohistochemistry (Rodda et al. 2018; Abe et al. 2022; Grasso et al. 2023).

### 2.1 LN FRCs

FRCs constitute about half of the nonimmune (CD45−) stromal cells within the LNs. They are crucial for creating a specialised network of conduit (pipe‐like) vessels embedded with extracellular matrix (ECM), that allows capture of antigens from the lymph fluid (Krishnamurty and Turley 2020; Grasso et al. 2021; Zou, Wiechers, and Huehn 2021; Chang and Turley 2015). This interconnected reticular network is rich in various growth factors and survival signals that support the trafficking, retention and function of various leukocytes. FRCs have the capability to present antigens, and are highly responsive to immune signals, growth factors and cytokines, making them central to maintaining a balance between immune tolerance and active defence in the LNs (Krishnamurty and Turley 2020; Grasso et al. 2021; Zou, Wiechers, and Huehn 2021; Chang and Turley 2015). During inflammation, FRCs undergo a series of changes—activation, stretching and proliferation—that enable LNs to expand and make room for increasing numbers of lymphocytes (Krishnamurty and Turley 2020; Grasso et al. 2021; Zou, Wiechers, and Huehn 2021; Chang and Turley 2015). Recent advances in single‐cell RNA (scRNA) sequencing have identified different subgroups of FRCs (Rodda et al. 2018; Abe et al. 2022; Grasso et al. 2023), each with distinct characteristics and roles within the LNs of both humans and mice (Table 1). This diversity highlights the complexity and specialised functions of FRCs in managing the immune environment.

**TABLE 1:** Identification of major LNSC subtypes.

Subtypes		Localisation	Unique transcriptomic signatures	Function	References
Fibroblastic reticular cells	Marginal reticular cells	Lining the subcapsular sinus (SCS)	PDPN+ MAdCAM1+ Bst1+ RANKL+ Cxcl13+ CD21/35−	Monitor and capture antigens from the SCS space. Support the survival of SCS macrophages via RANKL signalling. Maintain Type 3 innate lymphoid cells via IL7 signalling. MRC can differentiate into FDC during immune response.	Krishnamurty and Turley (2020), Grasso et al. (2021), Zou, Wiechers, and Huehn (2021), Rodda et al. (2018), Lütge et al. (2023)
	Follicular dendritic cells (FDCs)	B cell follicle and light zone of germinal centre	CD31− PDPN+ CD21/35+ MAdCAM1+/− Bst1+	Support B cell survival and maintenance via CXCL13 and BAFF. Present antigens to B cells via immunocomplexes bound to Fc‐receptors. Forms the light zone of GC where class‐switching and affinity maturation occurs.	Krishnamurty and Turley (2020), Grasso et al. (2021), Zou, Wiechers, and Huehn (2021), Rodda et al. (2018), Lütge et al. (2023)
	CXCL12+ follicular cells	Dark zone of germinal centre	CD21/35lo Baff+ Cxcl13− Cxcl12+	Forms the dark zone of the GC and attract CXCR4+ B cells for somatic hypermutation process.	Krishnamurty and Turley (2020), Grasso et al. (2021), Zou, Wiechers, and Huehn (2021), Rodda et al. (2018), Lütge et al. (2023)
	CCL19lo Ch25h+ TRC	Mainly at interfollicular (IF) region, T and B‐cell zone boundary	PDPN+ Ccl19lo, Ccl21+ Il7+ Bst1+ Cxcl13+ Baff+	Support T cell and B cell survival and maintenance, at the T/B borders and in the B cell follicles. Produce cholesterol‐25‐hydroxylase (Ch25h), critical for localisation of DC, T_FH_ and B cells to the follicle border.	Krishnamurty and Turley (2020), Grasso et al. (2021), Zou, Wiechers, and Huehn (2021), Rodda et al. (2018), Lütge et al. (2023)
	CCL19hi TRC	Primarily in T cell zone	PDPN+, Ccl19hi Ccl21+ Il7+ Bst1+	Support T cell and DCs survival and maintenance via IL‐7 and homeostatic chemokines.	Krishnamurty and Turley (2020), Grasso et al. (2021), Zou, Wiechers, and Huehn (2021), Rodda et al. (2018)
	CCL19lo IL7hi TRC	Boundary between T cell zone and medulla	PDPN+ Ccl19lo Ccl21+ Il7hi Bst1+ Cxcl12+ Baff+	Proposed to support homeostasis of B cells residing at this region.	Takeuchi et al. (2018, 2021)
	Medullary FRCs	Medulla	PDPN+ LepR+ BST1lo CXCL12hi	Medullary FRCs provide survival factors such as Il6, Tnfsf11, Cxcl13 and April for the maintenance of plasma cells.	Krishnamurty and Turley (2020), Grasso et al. (2021), Zou, Wiechers, and Huehn (2021), Rodda et al. (2018), Lütge et al. (2023)
	CD34+ FRC	Capsular area and surrounding large blood vessels in the medulla	PDPN+ CD34+	Serve as a progenitor for stromal cell development. May play a role in formation of new lymph and blood vessel. Facilitate LN expansion during immune response via CD248.	Krishnamurty and Turley (2020), Grasso et al. (2021), Zou, Wiechers, and Huehn (2021), Rodda et al. (2018), Lütge et al. (2023)
Lymphatic endothelial cells	SCS ceiling LEC	Lining the ceiling of SCS space	Lyve1+ Cav1+, Ackr4+	Regulate lymphocyte migration by scavenging CCL19 and CCL21 via expression of atypical chemokine receptor (Ackr4).	Krishnamurty and Turley (2020), Grasso et al. (2021), Zou, Wiechers, and Huehn (2021), Takeda et al. (2019), Fujimoto et al. (2020)
	SCS floor LEC	Lining the floor of SCS space	Lyve1+ Cd274hi Madcam1+ Ccl20+	Express high levels of PDL1, which may be involved in peripheral tolerance. Produces of CCL20 and able to attract CC6+ lymphocytes.	Krishnamurty and Turley (2020), Grasso et al. (2021), Zou, Wiechers, and Huehn (2021), Takeda et al. (2019), Fujimoto et al. (2020)
	Ptx3 LEC	Paracortical sinus lymphatics	Lyve1+ Ptx3+	Regulate lymphocyte egress from LNs.	Krishnamurty and Turley (2020), Grasso et al. (2021), Zou, Wiechers, and Huehn (2021), Takeda et al. (2019), Fujimoto et al. (2020)
	Macro LEC	Medullary sinus lymphatics	Lyve1+ Marco+ Cd209+	Marco LEC can recruit neutrophils and express a gene signature that supports the maintenance of innate immune response in the medullary region.	Krishnamurty and Turley (2020), Grasso et al. (2021), Zou, Wiechers, and Huehn (2021), Takeda et al. (2019), Fujimoto et al. (2020)
	Valve LEC	Throughout the lymphatic network	Lyve1+ Foxc2+ Cav1+ Esam+, Cldn11+	Prevent the backflow of lymph and facilitate the unidirectional flow of lymph though lymphatics.	Krishnamurty and Turley (2020), Grasso et al. (2021), Zou, Wiechers, and Huehn (2021), Takeda et al. (2019), Fujimoto et al. (2020)
Blood endothelial cells	Capillary BEC Type I	Throughout the lymph node	Igfbp3+ Col4a1+ Col4a2+	Regulation of the vascular tone and blood pressure.	Krishnamurty and Turley (2020), Grasso et al. (2021), Zou, Wiechers, and Huehn (2021), Brulois et al. (2020)
	Capillary BEC Type II		Erg1+ Cxcl1+, Sgk1+ Nr4a1+	Response to fluid laminar flow inside the vessels.	Krishnamurty and Turley (2020), Grasso et al. (2021), Zou, Wiechers, and Huehn (2021), Brulois et al. (2020)
	Transitional EC (TrEC)		Chst2+, St3Gal6+, Fut7+	Trajectory analyses suggest that TrECs might be a transitional phenotype during differentiation.	Krishnamurty and Turley (2020), Grasso et al. (2021), Zou, Wiechers, and Huehn (2021), Brulois et al. (2020)
	Capillary progenitors		Angpt2+ Apln+ Esm1+ Pgf+	CRPs might represent a population of precursor cells to maintain vascular cell homeostasis and vessel growth.	Krishnamurty and Turley (2020), Grasso et al. (2021), Zou, Wiechers, and Huehn (2021), Brulois et al. (2020)
	Arterial BEC		Cd31+, Gja4+, Gja5+, Bmx+, Sox17+, Gkn3	Arterial ECs express GJA5, GJA4, BMX and IGFBP3 which have a role in vascular tone and blood pressure.	Krishnamurty and Turley (2020), Grasso et al. (2021), Zou, Wiechers, and Huehn (2021), Brulois et al. (2020)
	High endothelial venule (HEV) BEC	Paracortex	Cd31+ PNAd+ Ccl21+ Chst4+ Glycam1+	HECs constitute the high endothelial venules where the HECs secrete Ccl21 to attract T cells, as well as expressing GlyCAM‐1, MadCAM1 and ICAM‐1 that allow recruitment of other lymphocytes.	Krishnamurty and Turley (2020), Grasso et al. (2021), Zou, Wiechers, and Huehn (2021), Brulois et al. (2020)
	Medullary BEC	Medulla	Ackr1+ Sele+ Selp+ Vcam1+	Medulla venule EC cells express the SELE and SELP genes which are involved in the regulation of neutrophil activation and platelet degranulation.	Krishnamurty and Turley (2020), Grasso et al. (2021), Zou, Wiechers, and Huehn (2021), Brulois et al. (2020)

### 2.2 LN LECs

Immune cells and antigens traverse the LNs through intricate subcapsular, cortical and medullary sinus networks, where LECs line the sinuses of the LN (Krishnamurty and Turley 2020; Grasso et al. 2021; Zou, Wiechers, and Huehn 2021; Chang and Turley 2015). Recent gene profiling studies of LN LECs have revealed distinct spatial and functional heterogeneity in subpopulations of LECs (Table 1; Takeda et al. 2019; Fujimoto et al. 2020; Xiang et al. 2020; den Braanker et al. 2021). For instance, LN floor LECs can act as a semipermeable barrier, and sort lymph‐borne antigens based of molecular sizes into the LN parenchyma. Floor LECs also secrete chemokines to control leukocyte trafficking within the LN. LN LECs are also reported for their ability to exert peripheral tolerance by expressing self‐antigens on MHC Class I and II molecules (Tewalt et al. 2012; Lucas and Tamburini 2019). During inflammation, activated LECs rapidly proliferate, expanding the lymphatic network to enhance entry of lymphocytes and dendritic cells (DC) into the LN.

### 2.3 LN BECs

BEC line the blood vessels that play a critical role in transporting soluble factors, regulating blood flow and recruitment of leukocytes into the LN. Recent scRNA‐seq studies have identified multiple BEC subsets (Table 1) (Abe et al. 2022; Brulois et al. 2020). With regard to leucocyte migration into the LNs, a specialised subset of BECs known as high endothelial venule (HEV) cells, are responsible for facilitating the entry of circulating leucocytes into the paracortex of the LN. HEV cells express Peripheral Node Addressin (PNAd), which is critical for lymphocyte trafficking, and the chemokines CCL21 and CCL19 to attract lymphocytes expressing CCR7, a cognate receptor for both CCL21 and CCL19 (Krishnamurty and Turley 2020; Grasso et al. 2021; Zou, Wiechers, and Huehn 2021; Chang and Turley 2015; Abe et al. 2022; Brulois et al. 2020). Another specialised subset of BECs in the LN medulla has been reported to preferentially mediate neutrophil trafficking into the LN during inflammation.

### 2.4 LN Pericytes and SMCs

LN pericytes and SMCs are a poorly characterised population of cells within the LN, partly because these groups are often analysed as a DN population. Malhotra et al. (2012) first identified this DN population as ‘pericytes’ due to their expression of Itga7, a canonical marker of pericytes. Through RNA‐seq studies, they observed that these ‘pericytes’ share similar properties with fibroblastic reticular cells (FRCs), such as the expression of chemokines like CCL21 and CCL19. A subsequent study by Sitnik et al. (2016) noted that the gene signatures of the DN subset included signatures suggestive of an SMC lineage (Sitnik et al. 2016). It was not until Abe et al. (2022) identified pericytes and SMCs as separate cell populations within the human LN. Given their close association with the LN vascular system, their function is likely related to the maintenance of vascular tone and blood vessel integrity (Bergers and Song 2005) within the LN.

## 3 Age‐Related Changes to the LN and LNSCs

### 3.1 Structural Disorganisation of the Aged LN

**FIGURE 1:**
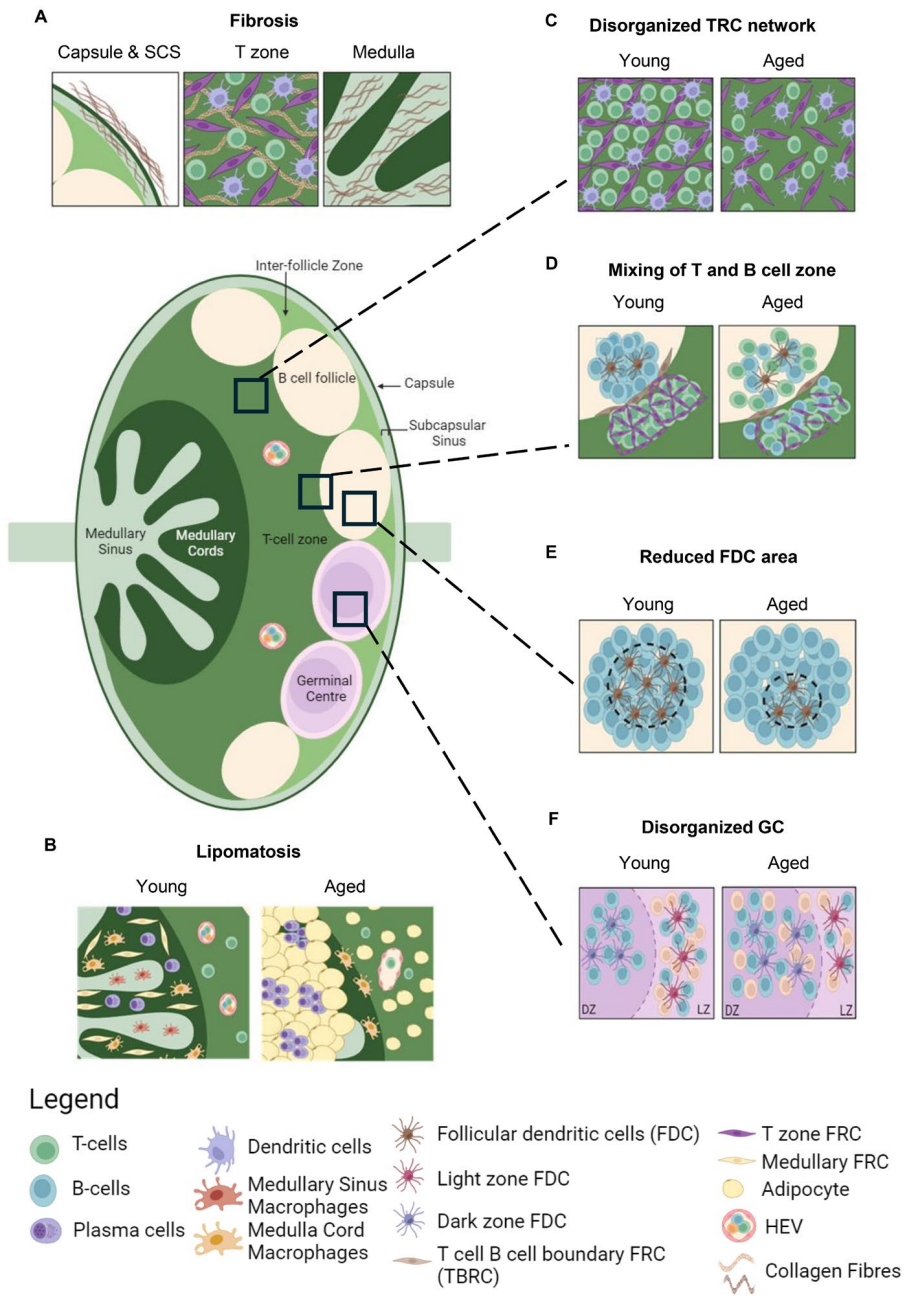
Age‐related changes in the stromal cells and lymph node structure and organisation. (A) Fibrosis is observed at the capsule and the sinus region of the LN, including the subcapsular sinus (SCS) and medullary sinus (MS). Fibrosis is also observed within the T cell zone and may impair the ability of T cell zone fibroblastic reticular cells (TRC) to maintain naïve T cells due to hinderance of bioavailability of trophic and homeostatic molecules such as IL‐7. (B) Fatty degeneration, also known as lipomatosis, is often observed starting at the MS, with gradually invasion into the T cell zone. The loss of the medullary cords (MC) leads to plasma cell being trapped within fatty deposits. Lipomatosis within the T cell zone also leads to irregular HEV morphology. (C) Loss of TRC network is observed in aged LN. The TRC network is reported to increase the frequency of DC‐T cell encounters leading to a faster selection of antigen‐specific T cells. Furthermore, TRC network stretching is important to increase the size of the LN during immune response to accommodate influx of lymphocytes. Loss of TRC network may impede important DC‐T cell interactions, impair LN swelling, leading to ineffective immune response. (D) Mixing of the T and B cell zone is often observed in aged LNs and is believed to be due to disruption of stromal cell network at the T‐B cell zone interface. Mixing of T‐B zones may impact the quality of the humoral immune responses. (E) FDC numbers decline with age, leading to reduced number of B cell follicles observed within the aged LN, and subsequently reducing number of GC during acute infection. (F) Dysregulation of chemokine with the GC of the LN leads to an expansion of the dark zone, characterised by mislocalisation of T cells, which impacts the quality of GC response during infection or vaccination, by reducing the number of affinity‐matured plasma cells (created with BioRender.com).

LN degeneration is a common phenomenon that becomes increasingly prevalent with age, affecting the structural organisation of LNs in various species, including humans, nonhuman primates and rodents (Luscieti et al. 1980; Jin et al. 2022; Pan, Suami, and Taylor 2008; Gödde et al. 2023). Histological examinations of LNs obtained from aged human individuals have revealed two main degenerative changes commonly observed in aged LNs: (1) Fibrosis, which includes the thickening of the capsule and trabeculae (absent in mice), replacement of smooth muscle cells by fibrous tissue (hyaline degeneration) and increased collagen fibres in various regions of the LN, including the cortex, medulla, sinuses and blood vessels (Figure 1A), and (2) lipomatosis, which is the gradual replacement of the LN with fat. Due to these degenerative changes, the LNs become devoid of immune cells (Figure 1B). Therefore, the presence of age‐associated lipomatosis and/or fibrosis is expected to impair the normal function of the LN.

In 2023, a study by Bekkhus et al. provided further insights into the consequences of LN lipomatosis (Bekkhus et al. 2023). The analysis of human LNs exhibiting different grades of lipomatosis revealed that lipomatosis often begins in the medullary region, followed by progressive invasion into the cortical region of the LN. Early lipomatosis is associated with extensive remodelling of the medullary region, including the loss of medullary sinus LECs and plasma cell disorganisation. Loss of medullary sinus LECs can impair efficient lymph drainage, while the consequences of plasma cell disorganisation remain to be elucidated. As lipomatosis progresses, HEVs become dilated, and in regions with dilated HEVs, a reduction in T cell density is observed, suggesting that lipomatosis can impact the recruitment of T cells into the LN. As a potential explanation for lipomatosis, it was shown that FRCs near the medullary region of human LNs with lipomatosis expressed both fibroblast (alpha‐smooth muscle actin) and adipocyte (perilipin) markers, suggesting a fibroblast‐to‐adipocyte transitional phenotype. FRCs residing in the medulla region belong to the CD34+ and BST1− subset (Table 1). Currently, it is not known what factors could induce their transformation into adipocytes during ageing. Elucidating the mechanism is particularly challenging because murine aged LNs rarely exhibit lipomatosis (Bekkhus et al. 2023). Choi et al. (2020) found that murine FRC precursors undergo adipocyte differentiation when deprived of lymphotoxin beta receptor (LTβR) mediated signalling. Further studies are required to determine whether the LTβR signalling axis is dysregulated during ageing and whether such dysregulation may precipitate the fibroblast‐to‐adipocyte transition in FRCs found in the medulla of human LNs.

Fibrosis is another degenerative change observed in aged LNs, and like lipomatosis, fibrotic changes may disrupt the proper functioning of immune cell niches within the LN, impeding efficient immune responses (Kityo et al. 2018). In 2022, Kwok et al. demonstrated that the movement of young, adoptively transferred naïve T cells is impaired in the T cell zone of aged LNs with increased collagen deposition (Kwok et al. 2022). Analysis of fibrotic LNs obtained from human immunodeficiency virus (HIV) infected patients or experimental models of simian immunodeficiency virus (SIV) infection indicated that fibrosis within the T cell zone led to a disrupted TRC network (Figure 1A,C), resulting in the depletion of T cells due to restricted access to IL‐7, a key T cell survival factor (Estes et al. 2007; Zeng et al. 2011). These studies shed light on how fibrosis may affect T cell niches and should encourage further research on how fibrotic changes may affect other immune cell niches within the aged LN. For instance, circulating B cells are also reported to migrate along the FRC network in the T cell zone to reach the follicles after exiting from the HEV (Bajénoff et al. 2006), and thus fibrosis may lead to improper localisation of B cells. At present, the mechanism of age‐associated LN fibrosis is unknown. Given that FRCs are the primary producer of collagen and other ECM fibres in the LN, their potential role in age‐associated LN fibrosis should be evaluated. In HIV/SIV infection (Zeng et al. 2011), skin transplant (Li, Zhao, et al. 2020) and injury to the kidney (Maarouf et al. 2018; Li et al. 2021), fibrosis of the draining LN was associated with increased collagen deposition, in which expression of collagen genes were upregulated in FRCs. Nevertheless, it is not known if the mechanism of age‐associated LN fibrosis is similar to that of chronic injury/infection‐induced fibrosis, which is that increased collagen production is the direct cause of fibrosis. Recent studies have highlighted that fibrosis may not be simply due to increased collagen production, but also an imbalance in collagen degradation, as well as changes in collagen structure and stability. For example, in a study to determine molecular programme of fibrotic changes in ageing human lungs, it was reported that there were no differences in expression of collagen genes between the young and the aged cohorts (Lee et al. 2021). Their study revealed that aged cohorts have higher expression of genes such as tissue inhibitors of matrix metalloproteases (TIMP), which inhibit collagen degradation and lysyl oxidases (LOX), transglutaminases (TGM), which could cause collagen cross‐linking, leading to increased tissue stiffness. The findings were also reported in ageing heart (Wu et al. 2020; Horn and Trafford 2016), and to some extent in the aged liver (Zhang et al. 2005), kidney (Hultström et al. 2012) and skeletal muscle (Chen, Lin, et al. 2021). Given data generated by Malhotra et al. have demonstrated FRCs are producers of matrix metalloproteinase (MMPs), TIMPs, LOX and TGM that suggest their role in the proper ECM maintenance of the LNs, it might be necessary to perform further studies to determine whether aged FRCs show any dysregulation in (1) ECM production, (2) ECM breakdown and (3) ECM modification, and how such dysregulation may precipitate into age‐induced fibrosis of the LN.

The mixing of T and B cell zones represents disorganisation of niches within the aged LNs (Figure 1D). With age, the boundary between the B cell follicles and T cell zones becomes irregular, with the B cell follicles becoming less distinct and more diffuse (Masters et al. 2019; Turner and Mabbott 2017). A study by Masters et al. (2019) observed that the mixing of T and B cell zones in aged LNs was associated with a disrupted stromal cell network at the T and B cell (T‐B) interface. In adult murine LNs, the T cell zone FRC (TRC) network clearly terminated before the B cell follicle. However, in aged LNs, the TRC network was observed to infiltrate the B cell follicle, leading to a disrupted B cell follicle architecture. A unique subset of FRCs, known as TBRCs, is reported to lay a reticular network at the T–B interface, with a distinctive chemokine signature of CCL21a and CXCL13 governing the proper positioning of T and B cells (Table 1; Rodda et al. 2018). It remains to be investigated whether any age‐associated changes to TBRCs may lead to the disrupted network at the T–B interface, resulting in the mixing of T and B cell zones.

### 3.2 Ageing of FRCs Impacts Diminished Functional Immunity to Infection and Vaccination

Remodelling of the LN stromal cell network is an important process during infection‐driven inflammation (Yang et al. 2014). To accommodate influx and subsequent lymphocyte proliferation, activated FRCs relaxes, leading to elongated stromal cell network and expansion of the LN. This remodelling process is mediated by interactions between FRCs and resident/migratory DCs (Acton et al. 2014; Astarita et al. 2015; de Winde et al. 2021). Recent analysis has demonstrated how the expansion of the LN is associated with improved immune responses (Najibi et al. 2024). While various studies have reported that aged FRCs exhibit reduced activation and delayed proliferative responses during infection (Masters et al. 2019; Denton et al. 2022; Bennett et al. 2023), it is not known how this may impair the expansion capacity of the LN. Masters et al. (2019) observed that despite delayed proliferation of aged FRCs during acute infection, there were no differences in the network length or branching points of the FRC network between aged and young LNs suggesting that the expansion of aged LN was not significantly affected.

Dysregulated expression of chemokines by aged FRCs also impacts T‐cell mediated immunity during response to pathogens. Thus far, majority of the studies have indicated that aged FRCs have reduced expression of either *Ccl19* or *Ccl21a* transcripts (Becklund et al. 2016; Kwok et al. 2022; Masters et al. 2019). During experimental West Nile virus (WNV) infection, the draining aged murine LN failed to upregulate CCL21a (Bennett et al. 2023), resulting in a decrease in recruitment of T cells and DCs. A decrease in the number of DCs can be detrimental during infection. Not only a reduced DC numbers may impair LN expansion due to less interactions with the FRCs, but it also impedes the delivery of processed antigens into the LN. Furthermore, activation of antigen‐specific T cells is also blocked due to lower co‐stimulatory signals derived from lesser number of DCs. The diminished activation of naïve T cells, compounded with their reduced migration into the inflamed LN, all acts towards the impairment to produce adequate antigen‐specific T cell for pathogen clearance (Masters et al. 2019).

To establish enduring humoral immunity, the generation of long‐lasting plasma cells capable of producing various antibody classes with high antigen affinity is crucial (Lee and Linterman 2022; Allen, Okada, and Cyster 2007). Within the GC, B cells, CD4+ follicular T helper cell (T_FH_) cells, macrophages and DC work collaboratively, connected by a stromal network formed by Follicular dendritic cells (FDCs). During infection, FDCs remodels the B cell follicle into distinct light zone and dark zone, favouring affinity selection and somatic hypermutation of GC B cells, which is necessary for plasma cell production (Lee and Linterman 2022; Wang et al. 2011). During ageing, a reduction in the proportion and numbers of FDCs, along with fewer and less densely packed GCs is observed in the LNs of aged rodents and human (Figure 1E; Luscieti et al. 1980; Silva‐Cayetano et al. 2023; Turner and Mabbott 2017; Stebegg et al. 2020). Intrinsically, aged FDCs display defective antigen presenting and co‐stimulatory capacity, leading to diminished antigen uptake and activation of B cells in the GC (Aydar et al. 2002, 2003). Recently, Silva‐Cayetano et al. (2023) reported that the organisation of the GC within the aged LN was altered after immunisation, characterised by a smaller light zone size and FDC network. Furthermore, they observed that aged T_FH_ cells are mislocalised to the dark zone of the GC in aged LNs, as opposed to their younger counterparts being confined to the light zone of the GC in adult murine LNs (Figure 1F). This mislocalisation led to an inadequate expansion of the FDC network and significantly impaired the quality of the antibody response (Silva‐Cayetano et al. 2023).

### 3.3 Age‐Associated Defects in Other LNSCs

Limited studies have impeded the understanding how ageing affects the other subset of LNSCs. Thus far, the only known defects reported so far are LECs and BECs having a reduced proliferation capacity during infection (Masters et al. 2019; Bennett et al. 2023) and HEVs exhibiting structural alterations, characterised by the loss of cuboidal morphology and thinning of vessel walls (Masters et al. 2019; Turner and Mabbott 2017). No studies thus far have characterised age‐associated defects in LN pericytes and SMCs.

Reflection from studies investigating age‐related changes in LECs from lymphatic vessels (LV) of rodents have revealed that with age, LV lose some of their ability to contract effectively, and their network becomes less dense (González‐Loyola and Petrova 2021; Kataru et al. 2022). Observance of increased permeability may be attributed to a reduction in glycocalyx thickness in endothelial cells and a loss of gap junction proteins. Such changes led to a decrease in the efficiency of local lymph drainage. Organ‐specific ageing of the lymphatic system is also observed in various studies. In aged LECs of the skin lymphatics, reduced expression of CCL21 was observed, indicating a potential impairment in attracting skin DCs to enter the LVs and migrate to the draining LN during infections (Kataru et al. 2022). In aged LECs of the meningeal lymphatics, reduced draining of cerebrospinal fluid was associated with increased risk of Alzheimer's disease (Rego, Sanchez, and Da Mesquita 2023).

A study performed by Chen, Sivan, et al. (2021) have demonstrated how the age loss in both BECs and pericytes are a common feature of tissue ageing (Chen, Sivan, et al. 2021). In their study, aged BECs in organs such as the brain, kidney and thymus demonstrated downregulation of vascular and pericyte maintenance pathways. The loss of pericytes lead to a reduced maintenance of blood vessel integrity and increased vascular leakiness, characterised by loss of ECM proteins. Disruption of vascular integrity is reported to be a factor in number of age‐associated diseases such as neurodegeneration (Berthiaume et al. 2022; Knox et al. 2022), retinal degeneration (Ehrlich et al. 2008) and kidney failure (Tanriover et al. 2023), substantiating how changes in the crosstalk between BECs and pericytes during ageing can lead to a loss of normal tissue function.

Given the evidence of how the ageing of lymphatic and vascular systems of other organs are detrimental to proper function, further studies are necessary to characterise age‐associated changes to LN LECs, BECs and pericytes, which are important cell types maintaining the lymphatic and vascular networks within the LN. These studies should investigate various functional parameters, including alterations (1) in chemokine and cytokine secretion, which have a potential impact on immune cell trafficking and activation, and (2) changes in ECM components that may impact the integrity of the lymphatic and vascular networks.

## 4 Potential Strategies to Improve the Function of Aged LNSCs

From the discussion above, it is evident that LNSCs undergo various age‐related changes that lead to structural and functional impairments in the LN. Finding ways that could temporarily enhancing or restoring the function of aged LNSCs, leading to improved immunity, could help to safeguard individuals over the age of 65 from the potential risks of future pandemics and other infectious diseases. Such strategies should be ideally widely accessible to the public.

### 4.1 Enhancing Aged LNSCs Activation During Vaccination

Currently, adjuvants are used to boost the effectiveness of vaccines by enhancing antigen‐specific humoral and cell‐mediated immune responses. Mechanisms of currently approved adjuvants involves activating pattern recognition receptors (PRRs), leading to enhance cytokine secretion (Reed, Orr, and Fox 2013; Pulendran, Arunachalam, and O'Hagan 2021). An example of PPRs is the Toll‐like receptors (TLRs), which recognises pathogen‐associated molecular patterns (PAMPs) such as lipopolysaccharide (LPS) or single‐stranded RNA (ssRNA) (Yang et al. 2022). In clinically approved vaccines, vaccines adjuvanted with TLR agonists such as CpG‐1018 (TLR‐9 agonist), monophosphoryl lipid A (MPL, TLR‐4 agonist), imiquimod (TLR7 agonist) have demonstrated efficacy in improving immune responses in older adults (Nanishi et al. 2022).

In 2022, Denton et al. reported that by injecting a GLA‐SE (TLR4 agonist) adjuvanted vaccine, the responses of marginal reticular cells (MRCs, expressing TLR4) in the aged LN were boosted, leading to improved GC responses, and slightly increasing the production of high affinity antibodies. This result suggests the feasibility of temporary enhancing activation of aged LNSC to improve immune responses in individuals over the age of 65 during vaccination (Denton et al. 2022). Expression of TLR3 on LNSCs has been documented (Fletcher et al. 2010), and thus using a TLR3 agonist as an adjuvant in vaccines may be a feasible option. Furthermore, with the widespread popularity of mRNA vaccines, using mRNA‐encoding proteins to temporary increase the activation of aged LNSCs is a viable strategy that warrants further investigation. Parallel to this idea, Brook et al. (2024), have recently demonstrated that the immune responses of aged mice can be improved by vaccines adjuvanted with mRNA encoding IL‐12p70, which acted to enhance the activation of aged DCs. In summary, the use of novel vaccines that can temporarily improve the function of aged LNSCs to enhance immune responses can be a readily clinical translatable implementation.

### 4.2 Senotherapeutics

Cellular senescence has been implicated in the pathophysiology of many age‐related diseases (Childs et al. 2015) and therefore reducing senescence burden through treatment with drugs selectively target senescent cells (SC) is one of the current focuses in gerontological research (Park and Shin 2022; Quarta and Demaria 2024). Therapeutic drugs targeting senescent cells can be broadly classified into two classes: (1) Senolytics—agents that induces apoptosis in SC and (2) Senomorphics—a wide range of agents that can modulate the phenotypes of SCs to those of young cells without induction of apoptosis. Senomorphics include antiageing/senescence compounds, such as telomerase activator, sirtuin activators, antioxidants and/or anti‐inflammatory agents (Kim and Kim 2019; Zhang et al. 2023).

While no studies have formally identified senescent LNSCs in the aged LNs, few studies have been undertaken to test the efficacy of senolytic agents in improving T and B cell‐mediated immunity in aged mice models of influenza or responses towards vaccinations. However, the results from these studies were mixed (Table 2; Camell et al. 2021; Lorenzo et al. 2022; Torrance et al. 2023; Cobanoglu et al. 2023). While no study thus far has addressed the usage of senomorphics in improving immune responses towards infection/vaccination in aged mice models, there are clinical trials investigating the efficacy of using senomorphics in improving immune responses in individuals over the age of 65 (Table 3; Mannick et al. 2014, 2018, 2021; Martin et al. 2023). At present, there is no clear consensus whether senomorphics or senolytics should be used. Therefore, more studies are required to be conducted to examine the efficacy of senotherapeutics as a method to improve and/or restore functional immunity in individuals over the age of 65, especially with regard to if senotherapeutics can be used to ameliorate or reverse the structural and functional defects of the aged LN stroma.

**TABLE 2:** Summary of studies using senotherapeutics to improve T and B cell responses in aged mice.

Study	Age of mice	Agents used	Senotherapeutic class	Experimental procedure	Outcome
Camell et al. (2021)	20 months	Fisetin	Senolytic	– Mice were exposed to soiled beddings from pet shop containing mouse hepatitis virus (MHV). – Mice were treated with 20 mg/kg/day Fisetin or vehicle by oral gavage daily for three consecutive days starting on Day 3 after exposure to MHV. The 3 days of treatment were repeated (3 days on, 4 days off) for 3 weeks. Animals were also fed standard chow with Fisetin added ad libitum after initiation of treatment.	– Aged mice treated with Fisetin showed improved survival over nontreated mice. – p16 and p21 expression, markers of senescence were decreased in major organs (e.g., spleen, liver). – Reduction in senescence‐associated secretory factors (SASP) factors (IL‐6, IL‐1 and CCL2). – Improved MHV antibody titres in treated age mice. – No mention of improvement in T cell mediated cellular response.
Lorenzo et al. (2022)	18–20 months	Dasatinib (D) + Quercetin (Q)	Senolytic	– Mice treated with 5 mg/kg/day of D and 50 mg/kg/day of Q via oral gavage. – Mice treated with intermittent dosing strategy of D + Q (treated for three consecutive days, rest 1 week, treated again for three consecutive days, rest 5 days before infection). – After D + Q treatment, mice were intranasally infected with sublethal dose of flu (H1N1 or H3N2) virus.	– Experiment(s) performed focused on CD4 differentiation and did not access if there were any changes to CD8 T cell responses and generation of flu specific antibodies. – D + Q led to a reduction of CD4 T_reg_ cells.
Torrance et al. (2023)	18–20 months	D + Q	Senolytic	– Mice treated with 5 mg/kg/day of D and 50 mg/kg/day of Q via oral gavage. – Mice treated with intermittent dosing strategy of D + Q (treated for three consecutive days, rest 1 week, treated again for three consecutive days, rest 5 days before infection). – After D + Q treatment, mice were intranasally infected with sublethal dose of flu (H1N1 or H3N2) virus.	– No improvement in survival outcome. – No reduction in SASP factors (IL‐6, TNF‐α, CCL2) in Bronchoalveolar lavage (BAL) after D + Q treatment. – No change in flu‐specific IgG antibodies between treatment and control. – Reduced but nonsignificant change in number of antigen‐specific CD8 T cells.
Cobanoglu et al. (2023)	22 months	ABT‐263	Senolytic	– Mice were treated by oral gavage (100 μL) with 50 mg/kg ABT‐263, or vehicle control administered for four consecutive days in two treatments. – Mice were then immunised with ovalbumin (OVA) subcutaneously.	– Reduced senescence cell burden in spleen. – Reduction in SASP factors (IL‐1β, IL‐6) production in aged splenocytes in ABT‐263 treated mice. – No change in OVA‐specific antibodies between treatment and control. – No change in OVA‐specific T cell responses (similar IFN‐γ responses).

**TABLE 3:** Clinical trials of using senomorphics in improving immune responses in individuals over the age of 65 years.

Study	Clinical trial number	Agents used	Clinical protocol	Outcome
Mannick et al. (2014)	ACTRN12611001241921	Everolimus (mTOR inhibitor)	– 218 adults aged ≥ 65 years were enrolled for study. – Patients were treated for 6 weeks with different doses of Everolimus, 0.5 mg daily, 5 mg weekly and 20 mg weekly or Placebo.	– Increase in antibody titres to influenza virus vaccine strains.
Mannick et al. (2018)	ACTRN12613001351707	Everolimus, BEZ235 (mTOR inhibitor)	– 264 adults aged ≥ 65 years. – Patients were treated with Everolimus, 0.1 mg daily, Everolimus, 0.5 mg daily, BEZ235, 10 mg daily, BEZ235, 10 mg + 0.1 mg everolimus daily or Placebo for 6 weeks.	– Increase in antibody titres to influenza virus vaccine strains. – Decreased infection rates in subjects aged 65 years and above during period of study.
Mannick et al. (2021)	Phase 2b ACTRN12617000468325 NCT03373903	BEZ235/Everolimus (mTOR inhibitor)	– 652 adults aged ≥ 65 years at increased risk of respiratory tract infections (RTI). – Patients treated with either BEZ235 (5 mg daily), BEZ235 (10 mg daily), BEZ235 (10 mg twice daily), BEZ235, 10 mg + everolimus, 0.1 mg daily or Placebo for 16 weeks.	– Decrease in incidence of laboratory‐confirmed RTIs.
	Phase 3 ACTRN12619000628145		– 1051 adults aged ≥ 65 years. – BEZ235, 10 mg daily or Placebo for 16 weeks.	– No decrease incidence in symptoms consistent with an RTI.
Martin et al. (2023)	NCT03996538	Metformin	– Nineteen healthy older adults (> 65) were enrolled in the study. – Patients were given escalating dose of metformin or placebo over 3 weeks: 500 mg metformin (placebo)/day for Week 1, 1000 mg metformin (placebo)/day for Week 2 and 1500 mg metformin (placebo)/day for Week 3. – 10 weeks after metformin/placebo treatment, patients were vaccinated with high‐dose flu vaccine.	– Metformin was well tolerated in patients with no signs of adverse events. – There were no differences in flu‐specific antibody titres, and flu‐specific T cell responses between patients receiving metformin or placebo. – Increased circulating T_FH_ were observed in patients receiving metformin. – Metformin reduced CD57 expression, a T cell senescence marker on CD4 T cell but not CD8 T cell. No change in exhaustion marker, PD‐1 observed in both CD4 and CD8 T cell. – Metformin did not alter levels of SASP such as IL‐6 and TNFα.

## 5 Questions in the Field

### 5.1 Elucidating Mechanisms of Ageing in LNSCs

Currently, the mechanisms behind the ageing of LNSCs remain unclear. Speculation points towards mechanisms such as cellular senescence, mitochondrial dysfunction and metabolic alterations (López‐Otín et al. 2023). Nevertheless, it is also likely that external factor may trigger and/or accelerate the manifestation of these ageing phenotypes (Bektas et al. 2018; Bachmann et al. 2020).

#### 5.1.1 Senescence of LNSCs and LN Ageing

Cellular senescence is a state of proliferation arrest of cells occurring during ageing. The persistence and accumulation of senescent cells has been demonstrated in various ageing studies and have implicated in the pathogenesis of many age‐related diseases. Thus, it is speculated that the senescence of LNSCs could be a potential mechanism behind LN ageing. The presence of senescent FRCs have been reported in draining LNs in models of skin transplant (Li, Zhao, et al. 2020) and injury to the kidney (Maarouf et al. 2018; Li et al. 2021). These senescent FRCs exhibited classical senescent phenotype, including upregulation of senescence‐associated Beta gal (SA‐β‐gal) and senescence genes such as *Cdkn2a* (p16) and *Cdkn1a* (p21). Senescent FRCs also produced senescence associated secreted phenotype (SASP) factors such as IL‐6 and transforming growth factor‐beta 1 (TGF‐β1), that led to a proinflammatory milieu and fibrogenic phenotype, respectively within the draining LNs. The similarity between the aged LNs and chronically inflamed LNs, such as onset of fibrosis suggest the presence of senescence FRCs that is yet to be identified. Future studies are required to identify the presence of senescence FRCs or LNSCs within the aged LNs, and how these population of cells contribute to LN ageing. Furthermore, the identification of these population of senescent cells is expected to potentiate future therapies involving senolytics/senomorphics to improve LN function in the older adults.

#### 5.1.2 Is Age Loss of Naïve T Cell an Initiating Factor of LN Ageing?

A hypothesis currently in the field suggest that the age loss of naïve T cells (T_N_ cells), due to thymus involution, is the driving cause of age‐associated LN atrophy (Sonar, Watanabe, and Nikolich 2023; Sonar et al. 2022). This hypothesis is currently driven by observation from studies where depletion of T_N_ cell in the LN results in disturbances to the stromal cell network and loss of immune cell niches within the LN, resembling an aged LN phenotype (Sonar, Watanabe, and Nikolich 2023; Zeng et al. 2012). However, it is uncertain if the results obtained from depletion studies, where T cells are suddenly removed are comparable to ageing, where T cells are gradually loss. Furthermore, in a study reported by Sonar et al. (2022), the authors monitored the trafficking of time‐stamped labelled recent thymic emigrants (RTEs) into LNs and discovered while the LN trafficking ability of aged murine RTEs is noninferior compared with its adult counterpart, due to early age‐associated changes in the superficial LNs, RTEs tend to exit and circulate to deeper LNs. Interpretation of this result suggest that atrophy of the LN may precede the loss of T_N_ cells, since preexisting structural defects such as fibrosis/lipomatosis and/or disrupted FRC network need to be present to prevent T_N_ cell seeding. Considering that there are existing mouse models that can attenuate thymus involution through overexpression of *Fgf21* (Youm et al. 2016), it would be valuable to use such models to investigate whether there is a causal relationship between loss of T_N_ cells and age‐associated LN atrophy.

#### 5.1.3 Circulatory and Local Mediators

Observations from experiments involving heterochronic (adult‐aged) parabiosis have suggested a possible role of circulatory factors that could induce LN atrophy (Davies et al. 2018). Davies et al. (2018) reported that in a pair of heterochronic parabiosed mice, there was a loss of naïve T cell maintenance and LNSCs in the LN of the adult parabiont, with numbers reduced to those seen in the old parabiont, suggesting that circulating factors present in the blood of the aged mouse, can result in the ageing of the young adult LN. Also, as LNs are stationed at different anatomic locations, local mediators derived from the tissue it drains may also have an impact in ageing of LNSCs (Cruz de Casas et al. 2024). Cruz de Casas et al. (2024) reviewed how each tissue draining LN is unique due to the composition and variety of immune cells, and exposure to tissue‐derived lymph, which contain several factors, such as cytokines, chemokines and metabolites. Hence it is conceivable that age‐associated changes to tissue‐derived lymph, could have a direct impact on the ageing of LNSCs in the corresponding draining LNs.

At present, the question here is to identify circulatory and/or local factors that may be pro‐LN ageing. These factors may be age‐associated increase in proinflammatory cytokines, such as tumour necrosis factor (TNF), IL‐6 or IL‐1, which are known to exert oxidative stress (Álvarez‐Rodríguez et al. 2012; Michaud et al. 2013; Baechle et al. 2023). Aged changes in the content of extracellular vesicles (EVs) may also be a factor (Takasugi 2018; Lananna and Imai 2021; Manni et al. 2023). Chen et al. (2024) have recently demonstrated that aged EVs carried microRNAs that could reduce the activity of peroxisome proliferator‐activated receptor (PPAR) γ coactivator α (PGC‐1α), a master regulator of mitochondrial biogenesis and function. The presence of these microRNAs was demonstrated to lead to mitochondrial dysfunction in hippocampus and muscles in vivo.

### 5.2 Does the Ageing of LNSCs Lead to the Onset of Age‐Associated Autoimmune Disorders?

Autoimmunity occurs when self‐reactive lymphocytes escape tolerance mechanisms and become activated in peripheral tissues, hence mechanisms are in place to prevent autoimmunity by the presentation of self‐antigens (Ring and Lakkis 1999; Romagnani 2006). It has been reported that LNSCs, including FRCs and LECs display unique expression patterns of self‐antigens on MHC‐II to induce tolerance in autoreactive T cells (Fletcher et al. 2010; Lee et al. 2007; Magnusson et al. 2008; Cohen et al. 2010). Mechanistically, self‐antigen presentation by LNSCs can convert CD4 T_N_ cells to regulatory T (T_REG_) cells and the continuous presentation of self‐antigens is important in regulating T_REG_ survival within the LN in vivo (Nadafi et al. 2020). The expression of peripheral tissue antigens (PTA) in LNSCs is mainly controlled by transcription factor: deformed epidermal autoregulatory factor 1 (DEAF1) (Fletcher et al. 2010). In experimental models of MHC‐II (Baptista et al. 2014; Dubrot et al. 2018) or DEAF1 (Yip et al. 2013) knockout in LNSCs, the frequency of autoreactive T cells is increased compared with controls, leading to the development of autoimmunity.

Studies have described the role of stromal‐derived laminins within the LN in regulating immunity and tolerance (Simon et al. 2019; Li, Shirkey, et al. 2020; Li et al. 2022). In LNs, the primary isoforms expressed by LNSCs are laminins α4β1γ1 and α5β1γ1. Immune responses have been associated with a reduced ratio of laminin α4 to α5, while tolerance has been linked to an elevated ratio. Depletion studies of α5 laminin led to greater numbers of Tregs in the T cell zone (Li, Shirkey, et al. 2020) while depletion studies of α4 laminin led to overactivation of T cells adopting an effector (Th1, Th2 and Th17) phenotype (Li et al. 2022). As such, it is possible that alterations in LN laminins, specifically, reduced laminin α4 to α5 ratio could play an associating factor in autoimmune disorder pathogenesis by driving Th17 immunity. Further studies are needed to prove this conjecture.

Given that many autoimmune conditions tend to manifest more frequently in the latter stages of adulthood (Amador‐Patarroyo, Rodriguez‐Rodriguez, and Montoya‐Ortiz 2012; Foley 2021), it remains unclear whether aged changes in LNSCs plays a role in this phenomenon. In vitro studies have demonstrated that aged murine FRCs are just as effective as their younger counterparts in inhibiting T cell activation through the production of nitric oxide (Masters et al. 2019). While this evidence argues against the notion that aged LNSCs contribute to the onset of autoimmune disorders, further investigation into other possible mechanisms, such as reduced expression of PTAs, MHC‐II or alterations in LN stroma laminins may provide insight into whether the ageing of LNSCs may lead to increased risk of autoimmune disorders.

## 6 Conclusion and Outlook

In recent years, there has been a noticeable increase in research activity within the field of lymphoid stromal cell immunology. Beyond their role in orchestrating immune responses following infection or vaccination, studies have also shed light on how LNSCs contribute to the induction of peripheral tolerance. Consequently, research into age‐related changes in LN stroma has gained increasing significance, where the goal is to elucidate the mechanisms responsible for the ageing of LNSC and how these age‐related changes might impact immune responses.

With much progress on the way in understanding aged changes of LNSCs in mice, one of the prominent challenges is the translatability of results obtained from murine studies to study age changes in human LNSCs. While current transcriptomic analyses have point towards phenotypic and functional similarity between murine and human LNSCs (Rodda et al. 2018; Abe et al. 2022; Takeda et al. 2019; Fujimoto et al. 2020; Xiang et al. 2020; Brulois et al. 2020; Kapoor et al. 2021), the presence of age‐associated lipomatosis in human LNs but not of murine LNs (Bekkhus et al. 2023) certainly suggest the presence of differences in age‐associated molecular changes between human LNSCs and murine LNSCs that requires further investigation.

It is anticipated that the field will make significant progress once scRNA‐seq datasets for aged murine LNSCs become available. Additionally, it is also hopeful that the Human Cell Atlas project (Rozenblatt‐Rosen et al. 2017) will lead the data collection effort from human LNs across various age groups. Consequently, the availability of data sets from murine and human studies could help researchers better characterise LNSCs beyond what has been currently established in the field. With more information at our disposal, conditional knockout or inducible gene overexpression models could be developed to address various questions in vitro and eventually in vivo. For instance, it is conceivable that the knockout of a specific stromal factor could lead to the premature ageing of the LN, or the constitutive expression of another stromal factor could potentially prevent LN ageing altogether. The outcome of such studies could facilitate the design of therapeutic strategies to target aged LNSCs, thereby restoring their function and improving immune outcomes for individuals over the age of 65 against infectious diseases and other age‐related immune dysregulation.

## Author Contributions

All authors contributed to the writing, revising and final approval of the manuscript.

## Conflicts of Interest

The authors declare no conflicts of interest.

## Data Availability

The authors have nothing to report.

## References

[acel70000-bib-0001] Abe, Y. , M. Sakata‐Yanagimoto , M. Fujisawa , et al. 2022. “A Single‐Cell Atlas of Non‐Haematopoietic Cells in Human Lymph Nodes and Lymphoma Reveals a Landscape of Stromal Remodelling.” Nature Cell Biology 24, no. 4: 565–578. 35332263 10.1038/s41556-022-00866-3 PMC9033586

[acel70000-bib-0002] Acton, S. E. , A. J. Farrugia , J. L. Astarita , et al. 2014. “Dendritic Cells Control Fibroblastic Reticular Network Tension and Lymph Node Expansion.” Nature 514, no. 7523: 498–502. 25341788 10.1038/nature13814 PMC4235005

[acel70000-bib-0003] Ahmadi, O. , J. L. McCall , and M. D. Stringer . 2013. “Does Senescence Affect Lymph Node Number and Morphology? A Systematic Review.” Australian and New Zealand Journal of Surgery 83, no. 9: 612–618. 10.1111/ans.12067 23347421

[acel70000-bib-0004] Allen, C. D. C. , T. Okada , and J. G. Cyster . 2007. “Germinal‐Center Organization and Cellular Dynamics.” Immunity 27, no. 2: 190–202. 17723214 10.1016/j.immuni.2007.07.009 PMC2242846

[acel70000-bib-0005] Álvarez‐Rodríguez, L. , M. López‐Hoyos , P. Muñoz‐Cacho , and V. M. Martínez‐Taboada . 2012. “Aging Is Associated With Circulating Cytokine Dysregulation.” Cellular Immunology 273, no. 2: 124–132. 22316526 10.1016/j.cellimm.2012.01.001

[acel70000-bib-0006] Amador‐Patarroyo, M. J. , A. Rodriguez‐Rodriguez , and G. Montoya‐Ortiz . 2012. “How Does Age at Onset Influence the Outcome of Autoimmune Diseases?” Autoimmune Diseases 2012: 251730. 22195277 10.1155/2012/251730 PMC3238350

[acel70000-bib-0007] Astarita, J. L. , V. Cremasco , J. Fu , et al. 2015. “The CLEC‐2‐Podoplanin Axis Controls the Contractility of Fibroblastic Reticular Cells and Lymph Node Microarchitecture.” Nature Immunology 16, no. 1: 75–84. 25347465 10.1038/ni.3035 PMC4270928

[acel70000-bib-0008] Aydar, Y. , P. Balogh , J. G. Tew , and A. K. Szakal . 2002. “Age‐Related Depression of FDC Accessory Functions and CD21 Ligand‐Mediated Repair of Co‐Stimulation.” European Journal of Immunology 32, no. 10: 2817–2826. 12355434 10.1002/1521-4141(2002010)32:10<2817::AID-IMMU2817>3.0.CO;2-Z

[acel70000-bib-0009] Aydar, Y. , P. Balogh , J. G. Tew , and A. K. Szakal . 2003. “Altered Regulation of Fc Gamma RII on Aged Follicular Dendritic Cells Correlates With Immunoreceptor Tyrosine‐Based Inhibition Motif Signaling in B Cells and Reduced Germinal Center Formation.” Journal of Immunology 171, no. 11: 5975–5987. 10.4049/jimmunol.171.11.5975 14634109

[acel70000-bib-0010] Bachmann, M. C. , S. Bellalta , R. Basoalto , et al. 2020. “The Challenge by Multiple Environmental and Biological Factors Induce Inflammation in Aging: Their Role in the Promotion of Chronic Disease.” Frontiers in Immunology 11: 570083. 33162985 10.3389/fimmu.2020.570083 PMC7591463

[acel70000-bib-0011] Baechle, J. J. , N. Chen , P. Makhijani , S. Winer , D. Furman , and D. A. Winer . 2023. “Chronic Inflammation and the Hallmarks of Aging.” Molecular Metabolism 74: 101755. 37329949 10.1016/j.molmet.2023.101755 PMC10359950

[acel70000-bib-0012] Bajénoff, M. , J. G. Egen , L. Y. Koo , et al. 2006. “Stromal Cell Networks Regulate Lymphocyte Entry, Migration, and Territoriality in Lymph Nodes.” Immunity 25, no. 6: 989–1001. 17112751 10.1016/j.immuni.2006.10.011 PMC2692293

[acel70000-bib-0013] Baptista, A. P. , R. Roozendaal , R. M. Reijmers , et al. 2014. “Lymph Node Stromal Cells Constrain Immunity via MHC Class II Self‐Antigen Presentation.” eLife 3: e04433. 25407678 10.7554/eLife.04433 PMC4270074

[acel70000-bib-0014] Becklund, B. R. , J. F. Purton , C. Ramsey , et al. 2016. “The Aged Lymphoid Tissue Environment Fails to Support Naïve T Cell Homeostasis.” Scientific Reports 6: 30842. 27480406 10.1038/srep30842 PMC4969611

[acel70000-bib-0015] Bekkhus, T. , A. Olofsson , Y. Sun , P. U. Magnusson , and M. H. Ulvmar . 2023. “Stromal Transdifferentiation Drives Lipomatosis and Induces Extensive Vascular Remodeling in the Aging Human Lymph Node.” Journal of Pathology 259, no. 3: 236–253. 36367235 10.1002/path.6030 PMC10108032

[acel70000-bib-0016] Bektas, A. , S. H. Schurman , R. Sen , and L. Ferrucci . 2018. “Aging, Inflammation and the Environment.” Experimental Gerontology 105: 10–18. 29275161 10.1016/j.exger.2017.12.015 PMC5909704

[acel70000-bib-0017] Bell, M. R. , and M. A. Kutzler . 2022. “An Old Problem With New Solutions: Strategies to Improve Vaccine Efficacy in the Elderly.” Advanced Drug Delivery Reviews 183: 114175. 35202770 10.1016/j.addr.2022.114175

[acel70000-bib-0018] Bennett, A. K. , M. Richner , M. D. Mun , and J. M. Richner . 2023. “Type I IFN Stimulates Lymph Node Stromal Cells From Adult and Old Mice During a West Nile Virus Infection.” Aging Cell 22, no. 4: e13796. 36802099 10.1111/acel.13796 PMC10086524

[acel70000-bib-0019] Bergers, G. , and S. Song . 2005. “The Role of Pericytes in Blood‐Vessel Formation and Maintenance.” Neuro‐Oncology 7, no. 4: 452–464. 16212810 10.1215/S1152851705000232 PMC1871727

[acel70000-bib-0020] Berthiaume, A.‐A. , F. Schmid , S. Stamenkovic , et al. 2022. “Pericyte Remodeling Is Deficient in the Aged Brain and Contributes to Impaired Capillary Flow and Structure.” Nature Communications 13, no. 1: 5912. 10.1038/s41467-022-33464-w PMC9547063 36207315

[acel70000-bib-0021] Brook, B. , V. Duval , S. Barman , et al. 2024. “Adjuvantation of a SARS‐CoV‐2 mRNA Vaccine With Controlled Tissue‐Specific Expression of an mRNA Encoding IL‐12p70.” Science Translational Medicine 16, no. 757: eadm8451. 39047117 10.1126/scitranslmed.adm8451

[acel70000-bib-0022] Brulois, K. , A. Rajaraman , A. Szade , et al. 2020. “A Molecular Map of Murine Lymph Node Blood Vascular Endothelium at Single Cell Resolution.” Nature Communications 11, no. 1: 3798. 10.1038/s41467-020-17291-5 PMC7393069 32732867

[acel70000-bib-0023] Camell, C. D. , M. J. Yousefzadeh , Y. Zhu , et al. 2021. “Senolytics Reduce Coronavirus‐Related Mortality in Old Mice.” Science 373, no. 6552: eabe4832. 34103349 10.1126/science.abe4832 PMC8607935

[acel70000-bib-0024] Chang, J. E. , and S. J. Turley . 2015. “Stromal Infrastructure of the Lymph Node and Coordination of Immunity.” Trends in Immunology 36, no. 1: 30–39. 25499856 10.1016/j.it.2014.11.003

[acel70000-bib-0025] Chen, J. , U. Sivan , S. L. Tan , et al. 2021. “High‐Resolution 3D Imaging Uncovers Organ‐Specific Vascular Control of Tissue Aging.” Science Advances 7, no. 6: eabd7819. 33536212 10.1126/sciadv.abd7819 PMC7857692

[acel70000-bib-0026] Chen, W.‐J. , I.‐H. Lin , C.‐W. Lee , and Y.‐F. Chen . 2021. “Aged Skeletal Muscle Retains the Ability to Remodel Extracellular Matrix for Degradation of Collagen Deposition After Muscle Injury.” International Journal of Molecular Sciences 22, no. 4: 2123. 33672763 10.3390/ijms22042123 PMC7924602

[acel70000-bib-0027] Chen, X. , Y. Luo , Q. Zhu , et al. 2024. “Small Extracellular Vesicles From Young Plasma Reverse Age‐Related Functional Declines by Improving Mitochondrial Energy Metabolism.” Nature Aging 4, no. 6: 814–838. 38627524 10.1038/s43587-024-00612-4 PMC11186790

[acel70000-bib-0028] Childs, B. G. , M. Durik , D. J. Baker , and J. M. van Deursen . 2015. “Cellular Senescence in Aging and Age‐Related Disease: From Mechanisms to Therapy.” Nature Medicine 21, no. 12: 1424–1435. 10.1038/nm.4000 PMC4748967 26646499

[acel70000-bib-0029] Choi, S. Y. , H. Bae , S. H. Jeong , et al. 2020. “YAP/TAZ Direct Commitment and Maturation of Lymph Node Fibroblastic Reticular Cells.” Nature Communications 11, no. 1: 519. 10.1038/s41467-020-14293-1 PMC6981200 31980640

[acel70000-bib-0030] Cobanoglu, O. , L. Delval , D. Ferrari , et al. 2023. “Depletion of Preexisting B‐Cell Lymphoma 2‐Expressing Senescent Cells Before Vaccination Impacts Antigen‐Specific Antitumor Immune Responses in Old Mice.” Aging Cell 22, no. 12: e14007. 37997569 10.1111/acel.14007 PMC10726819

[acel70000-bib-0031] Cohen, J. N. , C. J. Guidi , E. F. Tewalt , et al. 2010. “Lymph Node‐Resident Lymphatic Endothelial Cells Mediate Peripheral Tolerance via Aire‐Independent Direct Antigen Presentation.” Journal of Experimental Medicine 207, no. 4: 681–688. 20308365 10.1084/jem.20092465 PMC2856027

[acel70000-bib-0032] Cruz de Casas, P. , K. Knöpper , R. Dey Sarkar , and W. Kastenmüller . 2024. “Same Yet Different—How Lymph Node Heterogeneity Affects Immune Responses.” Nature Reviews Immunology 24, no. 5: 358–374. 10.1038/s41577-023-00965-8 38097778

[acel70000-bib-0033] Davies, J. S. , H. L. Thompson , V. Pulko , J. Padilla Torres , and J. Nikolich‐Žugich . 2018. “Role of Cell‐Intrinsic and Environmental Age‐Related Changes in Altered Maintenance of Murine T Cells in Lymphoid Organs.” Journals of Gerontology. Series A, Biological Sciences and Medical Sciences 73, no. 8: 1018–1026. 28582491 10.1093/gerona/glx102 PMC6037132

[acel70000-bib-0034] de Winde, C. M. , S. Makris , L. J. Millward , et al. 2021. “Fibroblastic Reticular Cell Response to Dendritic Cells Requires Coordinated Activity of Podoplanin, CD44 and CD9.” Journal of Cell Science 134, no. 14: jcs258610. 34184727 10.1242/jcs.258610 PMC8325952

[acel70000-bib-0035] den Braanker, H. , A. van Stigt , M. Kok , E. Lubberts , and R. Bisoendial . 2021. “Single‐Cell RNA Sequencing Reveals Heterogeneity and Functional Diversity of Lymphatic Endothelial Cells.” International Journal of Molecular Sciences 22, no. 21: 11976. 34769408 10.3390/ijms222111976 PMC8584409

[acel70000-bib-0036] Denton, A. E. , J. Dooley , I. Cinti , et al. 2022. “Targeting TLR4 During Vaccination Boosts MAdCAM‐1(+) Lymphoid Stromal Cell Activation and Promotes the Aged Germinal Center Response.” Science Immunology 7, no. 71: eabk0018. 35522725 10.1126/sciimmunol.abk0018 PMC7612953

[acel70000-bib-0037] Dubrot, J. , F. V. Duraes , G. Harlé , et al. 2018. “Absence of MHC‐II Expression by Lymph Node Stromal Cells Results in Autoimmunity.” Life Science Alliance 1, no. 6: e201800164. 30584641 10.26508/lsa.201800164 PMC6297861

[acel70000-bib-0038] Ehrlich, R. , A. Harris , N. S. Kheradiya , D. M. Winston , T. A. Ciulla , and B. Wirostko . 2008. “Age‐Related Macular Degeneration and the Aging Eye.” Clinical Interventions in Aging 3, no. 3: 473–482. 18982917 10.2147/cia.s2777 PMC2682379

[acel70000-bib-0039] Erofeeva, L. M. , and M. V. Mnikhovich . 2018. “Changes in the Structure and Cell Composition of Human Carinal Lymph Nodes During Aging.” Bulletin of Experimental Biology and Medicine 165, no. 5: 702–706. 30225704 10.1007/s10517-018-4246-z

[acel70000-bib-0040] Erofeeva, L. M. , and M. V. Mnikhovich . 2020. “Structural and Functional Changes in the Mesenteric Lymph Nodes in Humans During Aging.” Bulletin of Experimental Biology and Medicine 168, no. 5: 694–698. 32248450 10.1007/s10517-020-04782-0

[acel70000-bib-0041] Estes, J. D. , S. Wietgrefe , T. Schacker , et al. 2007. “Simian Immunodeficiency Virus‐Induced Lymphatic Tissue Fibrosis Is Mediated by Transforming Growth Factor β1‐Positive Regulatory T Cells and Begins in Early Infection.” Journal of Infectious Diseases 195, no. 4: 551–561. 17230415 10.1086/510852

[acel70000-bib-0042] Feehan, J. , N. Tripodi , and V. Apostolopoulos . 2021. “The Twilight of the Immune System: The Impact of Immunosenescence in Aging.” Maturitas 147: 7–13. 33832647 10.1016/j.maturitas.2021.02.006

[acel70000-bib-0043] Fletcher, A. L. , V. Lukacs‐Kornek , E. D. Reynoso , et al. 2010. “Lymph Node Fibroblastic Reticular Cells Directly Present Peripheral Tissue Antigen Under Steady‐State and Inflammatory Conditions.” Journal of Experimental Medicine 207, no. 4: 689–697. 20308362 10.1084/jem.20092642 PMC2856033

[acel70000-bib-0044] Foley, J. F. 2021. “Aging and Autoimmunity.” Science Signaling 14, no. 679: eabj0430.

[acel70000-bib-0045] Fujimoto, N. , Y. He , M. D'Addio , C. Tacconi , M. Detmar , and L. C. Dieterich . 2020. “Single‐Cell Mapping Reveals New Markers and Functions of Lymphatic Endothelial Cells in Lymph Nodes.” PLoS Biology 18, no. 4: e3000704. 32251437 10.1371/journal.pbio.3000704 PMC7162550

[acel70000-bib-0046] Fulop, T. , J. McElhaney , G. Pawelec , et al. 2015. “Frailty, Inflammation and Immunosenescence.” Interdisciplinary Topics in Gerontology 41: 26–40. 10.1159/000381134 26301977

[acel70000-bib-0047] Gödde, D. , S. Degener , C. Walles , et al. 2023. “Degenerative Changes in Aging Human Pelvic Lymph Nodes—A Reason to Rethink Staging and Therapy of Regional Malignancies?” Cancers (Basel) 15, no. 19: 4754. 37835449 10.3390/cancers15194754 PMC10571730

[acel70000-bib-0048] González‐Loyola, A. , and T. V. Petrova . 2021. “Development and Aging of the Lymphatic Vascular System.” Advanced Drug Delivery Reviews 169: 63–78. 33316347 10.1016/j.addr.2020.12.005

[acel70000-bib-0049] Goronzy, J. J. , F. Fang , M. M. Cavanagh , Q. Qi , and C. M. Weyand . 2015. “Naive T Cell Maintenance and Function in Human Aging.” Journal of Immunology 194, no. 9: 4073–4080. 10.4049/jimmunol.1500046 PMC4452284 25888703

[acel70000-bib-0050] Grasso, C. , C. Pierie , R. E. Mebius , and L. G. M. van Baarsen . 2021. “Lymph Node Stromal Cells: Subsets and Functions in Health and Disease.” Trends in Immunology 42, no. 10: 920–936. 34521601 10.1016/j.it.2021.08.009

[acel70000-bib-0051] Grasso, C. , J. Roet , C. G. de Graça , et al. 2023. “Identification and Mapping of Human Lymph Node Stromal Cell Subsets by Combining Single‐Cell RNA Sequencing With Spatial Transcriptomics.” *bioRxiv*. 10.1101/2023.08.18.553530. PMC12154172 40498289

[acel70000-bib-0052] Hadamitzky, C. , H. Spohr , A. S. Debertin , S. Guddat , M. Tsokos , and R. Pabst . 2010. “Age‐Dependent Histoarchitectural Changes in Human Lymph Nodes: An Underestimated Process With Clinical Relevance?” Journal of Anatomy 216, no. 5: 556–562. 20345860 10.1111/j.1469-7580.2010.01213.x PMC2871991

[acel70000-bib-0053] Hamazaki, Y. , M. Sekai , and N. Minato . 2016. “Medullary Thymic Epithelial Stem Cells: Role in Thymic Epithelial Cell Maintenance and Thymic Involution.” Immunological Reviews 271, no. 1: 38–55. 27088906 10.1111/imr.12412

[acel70000-bib-0054] Hazeldine, J. , and J. M. Lord . 2013. “The Impact of Ageing on Natural Killer Cell Function and Potential Consequences for Health in Older Adults.” Ageing Research Reviews 12, no. 4: 1069–1078. 23660515 10.1016/j.arr.2013.04.003 PMC4147963

[acel70000-bib-0055] Ho, Y. H. , and S. Méndez‐Ferrer . 2020. “Microenvironmental Contributions to Hematopoietic Stem Cell Aging.” Haematologica 105, no. 1: 38–46. 31806690 10.3324/haematol.2018.211334 PMC6939521

[acel70000-bib-0056] Horn, M. A. , and A. W. Trafford . 2016. “Aging and the Cardiac Collagen Matrix: Novel Mediators of Fibrotic Remodelling.” Journal of Molecular and Cellular Cardiology 93: 175–185. 26578393 10.1016/j.yjmcc.2015.11.005 PMC4945757

[acel70000-bib-0057] Hultström, M. , S. Leh , A. Paliege , S. Bachmann , T. Skogstrand , and B. M. Iversen . 2012. “Collagen‐Binding Proteins in Age‐Dependent Changes in Renal Collagen Turnover: Microarray Analysis of mRNA Expression.” Physiological Genomics 44, no. 10: 576–586. 22454451 10.1152/physiolgenomics.00186.2011

[acel70000-bib-0058] Jin, Z.‐W. , M. Aoki , K. Ueda , et al. 2022. “Human Lymph Node Degeneration in the Thoracic Region: A Morphometric and Immunohistochemical Analysis Using Surgically Obtained Specimens.” Frontiers in Physiology 13: 990801. 36187759 10.3389/fphys.2022.990801 PMC9515507

[acel70000-bib-0059] Kapoor, V. N. , S. Müller , S. Keerthivasan , et al. 2021. “Gremlin 1(+) Fibroblastic Niche Maintains Dendritic Cell Homeostasis in Lymphoid Tissues.” Nature Immunology 22, no. 5: 571–585. 33903764 10.1038/s41590-021-00920-6

[acel70000-bib-0060] Kataru, R. P. , H. J. Park , J. Shin , et al. 2022. “Structural and Functional Changes in Aged Skin Lymphatic Vessels.” Frontiers in Aging 3: 864860. 35821848 10.3389/fragi.2022.864860 PMC9261401

[acel70000-bib-0061] Kim, E. C. , and J. R. Kim . 2019. “Senotherapeutics: Emerging Strategy for Healthy Aging and Age‐Related Disease.” BMB Reports 52, no. 1: 47–55. 30526770 10.5483/BMBRep.2019.52.1.293 PMC6386227

[acel70000-bib-0062] Kityo, C. , K. N. Makamdop , M. Rothenberger , et al. 2018. “Lymphoid Tissue Fibrosis Is Associated With Impaired Vaccine Responses.” Journal of Clinical Investigation 128, no. 7: 2763–2773. 29781814 10.1172/JCI97377 PMC6025977

[acel70000-bib-0063] Knox, E. G. , M. R. Aburto , G. Clarke , J. F. Cryan , and C. M. O'Driscoll . 2022. “The Blood‐Brain Barrier in Aging and Neurodegeneration.” Molecular Psychiatry 27, no. 6: 2659–2673. 35361905 10.1038/s41380-022-01511-z PMC9156404

[acel70000-bib-0064] Kousa, A. I. , L. Jahn , K. Zhao , et al. 2024. “Age‐Related Epithelial Defects Limit Thymic Function and Regeneration.” Nature Immunology 25: 1593–1606. 39112630 10.1038/s41590-024-01915-9 PMC11362016

[acel70000-bib-0065] Krishnamurty, A. T. , and S. J. Turley . 2020. “Lymph Node Stromal Cells: Cartographers of the Immune System.” Nature Immunology 21, no. 4: 369–380. 32205888 10.1038/s41590-020-0635-3

[acel70000-bib-0066] Kwok, T. , S. C. Medovich , I. A. Silva‐Junior , et al. 2022. “Age‐Associated Changes to Lymph Node Fibroblastic Reticular Cells.” Frontiers in Aging 3: 838943. 35821826 10.3389/fragi.2022.838943 PMC9261404

[acel70000-bib-0067] Lananna, B. V. , and S. I. Imai . 2021. “Friends and Foes: Extracellular Vesicles in Aging and Rejuvenation.” FASEB Bioadvances 3, no. 10: 787–801. 34632314 10.1096/fba.2021-00077 PMC8493967

[acel70000-bib-0068] Lancaster, J. N. 2023. “Aging of Lymphoid Stromal Architecture Impacts Immune Responses.” Seminars in Immunology 70: 101817. 37572552 10.1016/j.smim.2023.101817 PMC10929705

[acel70000-bib-0069] Lee, J. L. , and M. A. Linterman . 2022. “Mechanisms Underpinning Poor Antibody Responses to Vaccines in Ageing.” Immunology Letters 241: 1–14. 34767859 10.1016/j.imlet.2021.11.001 PMC8765414

[acel70000-bib-0070] Lee, J. W. , M. Epardaud , J. Sun , et al. 2007. “Peripheral Antigen Display by Lymph Node Stroma Promotes T Cell Tolerance to Intestinal Self.” Nature Immunology 8, no. 2: 181–190. 17195844 10.1038/ni1427

[acel70000-bib-0071] Lee, S. , M. N. Islam , K. Boostanpour , et al. 2021. “Molecular Programs of Fibrotic Change in Aging Human Lung.” Nature Communications 12, no. 1: 6309. 10.1038/s41467-021-26603-2 PMC8563941 34728633

[acel70000-bib-0072] Li, L. , M. W. Shirkey , T. Zhang , et al. 2020. “The Lymph Node Stromal Laminin α5 Shapes Alloimmunity.” Journal of Clinical Investigation 130, no. 5: 2602–2619. 32017712 10.1172/JCI135099 PMC7190966

[acel70000-bib-0073] Li, L. , M. W. Shirkey , T. Zhang , et al. 2022. “Lymph Node Fibroblastic Reticular Cells Preserve a Tolerogenic Niche in Allograft Transplantation Through Laminin α4.” Journal of Clinical Investigation 132, no. 13: e156994. 35775481 10.1172/JCI156994 PMC9246384

[acel70000-bib-0074] Li, X. , J. Zhao , S. M. Naini , et al. 2021. “Kidney‐Draining Lymph Node Fibrosis Following Unilateral Ureteral Obstruction.” Frontiers in Immunology 12: 768412. 35024041 10.3389/fimmu.2021.768412 PMC8744208

[acel70000-bib-0075] Li, X. , J. Zhao , V. Kasinath , et al. 2020. “Lymph Node Fibroblastic Reticular Cells Deposit Fibrosis‐Associated Collagen Following Organ Transplantation.” Journal of Clinical Investigation 130, no. 8: 4182–4194. 32597832 10.1172/JCI136618 PMC7410068

[acel70000-bib-0076] Liang, Z. , X. Dong , Z. Zhang , Q. Zhang , and Y. Zhao . 2022. “Age‐Related Thymic Involution: Mechanisms and Functional Impact.” Aging Cell 21, no. 8: e13671. 35822239 10.1111/acel.13671 PMC9381902

[acel70000-bib-0077] López‐Otín, C. , M. A. Blasco , L. Partridge , M. Serrano , and G. Kroemer . 2023. “Hallmarks of Aging: An Expanding Universe.” Cell 186, no. 2: 243–278. 36599349 10.1016/j.cell.2022.11.001

[acel70000-bib-0078] Lorenzo, E. C. , B. L. Torrance , S. R. Keilich , et al. 2022. “Senescence‐Induced Changes in CD4 T Cell Differentiation Can Be Alleviated by Treatment With Senolytics.” Aging Cell 21, no. 1: e13525. 34962049 10.1111/acel.13525 PMC8761018

[acel70000-bib-0079] Lucas, E. D. , and B. A. J. Tamburini . 2019. “Lymph Node Lymphatic Endothelial Cell Expansion and Contraction and the Programming of the Immune Response.” Frontiers in Immunology 10: 36. 30740101 10.3389/fimmu.2019.00036 PMC6357284

[acel70000-bib-0080] Luscieti, P. , T. Hubschmid , H. Cottier , M. W. Hess , and L. H. Sobin . 1980. “Human Lymph Node Morphology as a Function of Age and Site.” Journal of Clinical Pathology 33, no. 5: 454–461. 7400343 10.1136/jcp.33.5.454 PMC1146110

[acel70000-bib-0081] Lütge, M. , A. de Martin , C. Gil‐Cruz , et al. 2023. “Conserved Stromal–Immune Cell Circuits Secure B Cell Homeostasis and Function.” Nature Immunology 24, no. 7: 1149–1160. 37202489 10.1038/s41590-023-01503-3 PMC10307622

[acel70000-bib-0082] Maarouf, O. H. , M. Uehara , V. Kasinath , et al. 2018. “Repetitive Ischemic Injuries to the Kidneys Result in Lymph Node Fibrosis and Impaired Healing.” Journal of Clinical Investigation Insight 3, no. 13: e120546. 10.1172/jci.insight.120546. 29997302 PMC6124521

[acel70000-bib-0083] Magnusson, F. C. , R. S. Liblau , H. von Boehmer , et al. 2008. “Direct Presentation of Antigen by Lymph Node Stromal Cells Protects Against CD8 T‐Cell‐Mediated Intestinal Autoimmunity.” Gastroenterology 134, no. 4: 1028–1037. 18395084 10.1053/j.gastro.2008.01.070

[acel70000-bib-0084] Malhotra, D. , A. L. Fletcher , J. Astarita , et al. 2012. “Transcriptional Profiling of Stroma From Inflamed and Resting Lymph Nodes Defines Immunological Hallmarks.” Nature Immunology 13, no. 5: 499–510. 22466668 10.1038/ni.2262 PMC3366863

[acel70000-bib-0085] Manni, G. , S. Buratta , M. T. Pallotta , et al. 2023. “Extracellular Vesicles in Aging: An Emerging Hallmark?” Cells 12, no. 4: 527. 36831194 10.3390/cells12040527 PMC9954704

[acel70000-bib-0086] Mannick, J. B. , G. del Giudice , M. Lattanzi , et al. 2014. “mTOR Inhibition Improves Immune Function in the Elderly.” Science Translational Medicine 6, no. 268: 268ra179. 10.1126/scitranslmed.3009892 25540326

[acel70000-bib-0087] Mannick, J. B. , M. Morris , H. U. P. Hockey , et al. 2018. “TORC1 Inhibition Enhances Immune Function and Reduces Infections in the Elderly.” Science Translational Medicine 10, no. 449: eaaq1564. 29997249 10.1126/scitranslmed.aaq1564

[acel70000-bib-0088] Mannick, J. B. , G. Teo , P. Bernardo , et al. 2021. “Targeting the Biology of Ageing With mTOR Inhibitors to Improve Immune Function in Older Adults: Phase 2b and Phase 3 Randomised Trials.” Lancet Healthy Longevity 2, no. 5: e250–e262. 33977284 10.1016/S2666-7568(21)00062-3 PMC8102040

[acel70000-bib-0089] Martin, D. E. , A. N. Cadar , H. Panier , B. L. Torrance , G. A. Kuchel , and J. M. Bartley . 2023. “The Effect of Metformin on Influenza Vaccine Responses in Nondiabetic Older Adults: A Pilot Trial.” Immunity & Ageing 20, no. 1: 18. 37131271 10.1186/s12979-023-00343-x PMC10152024

[acel70000-bib-0090] Masters, A. R. , A. Hall , J. M. Bartley , et al. 2019. “Assessment of Lymph Node Stromal Cells as an Underlying Factor in Age‐Related Immune Impairment.” Journals of Gerontology. Series A, Biological Sciences and Medical Sciences 74, no. 11: 1734–1743. 30721932 10.1093/gerona/glz029 PMC6777091

[acel70000-bib-0091] Masters, A. R. , L. Haynes , D. M. Su , and D. B. Palmer . 2017. “Immune Senescence: Significance of the Stromal Microenvironment.” Clinical and Experimental Immunology 187, no. 1: 6–15. 27529161 10.1111/cei.12851 PMC5167042

[acel70000-bib-0092] Michaud, M. , L. Balardy , G. Moulis , et al. 2013. “Proinflammatory Cytokines, Aging, and Age‐Related Diseases.” Journal of the American Medical Directors Association 14, no. 12: 877–882. 23792036 10.1016/j.jamda.2013.05.009

[acel70000-bib-0093] Montgomery, R. R. , and A. C. Shaw . 2015. “Paradoxical Changes in Innate Immunity in Aging: Recent Progress and New Directions.” Journal of Leukocyte Biology 98, no. 6: 937–943. 26188078 10.1189/jlb.5MR0315-104R PMC4661037

[acel70000-bib-0094] Morrisette‐Thomas, V. , A. A. Cohen , T. Fülöp , et al. 2014. “Inflamm‐Aging Does Not Simply Reflect Increases in Pro‐Inflammatory Markers.” Mechanisms of Ageing and Development 139: 49–57. 25011077 10.1016/j.mad.2014.06.005 PMC5881904

[acel70000-bib-0095] Nadafi, R. , C. Gago de Graça , E. D. Keuning , et al. 2020. “Lymph Node Stromal Cells Generate Antigen‐Specific Regulatory T Cells and Control Autoreactive T and B Cell Responses.” Cell Reports 30, no. 12: 4110–4123.e4. 32209472 10.1016/j.celrep.2020.03.007

[acel70000-bib-0096] Naismith, E. , and L. Pangrazzi . 2019. “The Impact of Oxidative Stress, Inflammation, and Senescence on the Maintenance of Immunological Memory in the Bone Marrow in Old Age.” Bioscience Reports 39, no. 5: BSR20190371. 31018996 10.1042/BSR20190371 PMC6522741

[acel70000-bib-0097] Najibi, A. J. , R. S. Lane , M. C. Sobral , et al. 2024. “Durable Lymph‐Node Expansion Is Associated With the Efficacy of Therapeutic Vaccination.” Nature Biomedical Engineering 8, no. 10: 1226–1242. 10.1038/s41551-024-01209-3. PMC11485260 38710838

[acel70000-bib-0098] Nanishi, E. , A. Angelidou , C. Rotman , D. J. Dowling , O. Levy , and A. Ozonoff . 2022. “Precision Vaccine Adjuvants for Older Adults: A Scoping Review.” Clinical Infectious Diseases 75, no. S1: S72–S80. 35439286 10.1093/cid/ciac302 PMC9376277

[acel70000-bib-0099] Osterholm, M. T. , N. S. Kelley , A. Sommer , and E. A. Belongia . 2012. “Efficacy and Effectiveness of Influenza Vaccines: A Systematic Review and Meta‐Analysis.” Lancet Infectious Diseases 12, no. 1: 36–44. 22032844 10.1016/S1473-3099(11)70295-X

[acel70000-bib-0100] Pan, W. R. , H. Suami , and G. I. Taylor . 2008. “Senile Changes in Human Lymph Nodes.” Lymphatic Research and Biology 6, no. 2: 77–83. 18564922 10.1089/lrb.2007.1023

[acel70000-bib-0101] Pangrazzi, L. , A. Meryk , E. Naismith , et al. 2017. “‘Inflamm‐Aging’ Influences Immune Cell Survival Factors in Human Bone Marrow.” European Journal of Immunology 47, no. 3: 481–492. 27995612 10.1002/eji.201646570 PMC5434810

[acel70000-bib-0102] Park, J. , and D. W. Shin . 2022. “Senotherapeutics and Their Molecular Mechanism for Improving Aging.” Biomolecules & Therapeutics 30, no. 6: 490–500. 36226551 10.4062/biomolther.2022.114 PMC9622307

[acel70000-bib-0103] Pritz, T. , B. Weinberger , and B. Grubeck‐Loebenstein . 2014. “The Aging Bone Marrow and Its Impact on Immune Responses in Old Age.” Immunology Letters 162, no. 1 pt. B: 310–315. 25014741 10.1016/j.imlet.2014.06.016

[acel70000-bib-0104] Pulendran, B. , P. A. Arunachalam , and D. T. O'Hagan . 2021. “Emerging Concepts in the Science of Vaccine Adjuvants.” Nature Reviews Drug Discovery 20, no. 6: 454–475. 33824489 10.1038/s41573-021-00163-y PMC8023785

[acel70000-bib-0105] Quarta, M. , and M. Demaria . 2024. “On the Past, Present and Future of Senotherapeutics.” NPJ Aging 10, no. 1: 11. 38310117 10.1038/s41514-024-00139-3 PMC10838299

[acel70000-bib-0106] Reed, S. G. , M. T. Orr , and C. B. Fox . 2013. “Key Roles of Adjuvants in Modern Vaccines.” Nature Medicine 19, no. 12: 1597–1608. 10.1038/nm.3409 24309663

[acel70000-bib-0107] Rego, S. , G. Sanchez , and S. Da Mesquita . 2023. “Current Views on Meningeal Lymphatics and Immunity in Aging and Alzheimer's Disease.” Molecular Neurodegeneration 18, no. 1: 55. 37580702 10.1186/s13024-023-00645-0 PMC10424377

[acel70000-bib-0108] Richner, J. M. , G. B. Gmyrek , J. Govero , et al. 2015. “Age‐Dependent Cell Trafficking Defects in Draining Lymph Nodes Impair Adaptive Immunity and Control of West Nile Virus Infection.” PLoS Pathogens 11, no. 7: e1005027. 26204259 10.1371/journal.ppat.1005027 PMC4512688

[acel70000-bib-0109] Ring, G. H. , and F. G. Lakkis . 1999. “Breakdown of Self‐Tolerance and the Pathogenesis of Autoimmunity.” Seminars in Nephrology 19, no. 1: 25–33. 9952278

[acel70000-bib-0110] Rodda, L. B. , E. Lu , M. L. Bennett , et al. 2018. “Single‐Cell RNA Sequencing of Lymph Node Stromal Cells Reveals Niche‐Associated Heterogeneity.” Immunity 48, no. 5: 1014–1028.e6. 29752062 10.1016/j.immuni.2018.04.006 PMC5971117

[acel70000-bib-0111] Romagnani, S. 2006. “Immunological Tolerance and Autoimmunity.” Internal and Emergency Medicine 1, no. 3: 187–196. 17120464 10.1007/BF02934736

[acel70000-bib-0112] Rozenblatt‐Rosen, O. , M. J. T. Stubbington , A. Regev , and S. A. Teichmann . 2017. “The Human Cell Atlas: From Vision to Reality.” Nature 550, no. 7677: 451–453. 29072289 10.1038/550451a

[acel70000-bib-0113] Silva‐Cayetano, A. , S. Fra‐Bido , P. A. Robert , et al. 2023. “Spatial Dysregulation of T Follicular Helper Cells Impairs Vaccine Responses in Aging.” Nature Immunology 24, no. 7: 1124–1137. 37217705 10.1038/s41590-023-01519-9 PMC10307630

[acel70000-bib-0114] Simon, T. , L. Li , C. Wagner , et al. 2019. “Differential Regulation of T‐Cell Immunity and Tolerance by Stromal Laminin Expressed in the Lymph Node.” Transplantation 103, no. 10: 2075–2089. 31343575 10.1097/TP.0000000000002774 PMC6768765

[acel70000-bib-0143] Sitnik, K. M. , K. Wendland , H. Weishaupt , et al. 2016. “Context‐Dependent Development of Lymphoid Stroma From Adult CD34(+) Adventitial Progenitors.” Cell reports 14, no. 10: 2375–2388. 26947077 10.1016/j.celrep.2016.02.033

[acel70000-bib-0115] Solana, R. , R. Tarazona , I. Gayoso , O. Lesur , G. Dupuis , and T. Fulop . 2012. “Innate Immunosenescence: Effect of Aging on Cells and Receptors of the Innate Immune System in Humans.” Seminars in Immunology 24, no. 5: 331–341. 22560929 10.1016/j.smim.2012.04.008

[acel70000-bib-0116] Sonar, S. A. , J. L. Uhrlaub , C. P. Coplen , et al. 2022. “Early Age‐Related Atrophy of Cutaneous Lymph Nodes Precipitates an Early Functional Decline in Skin Immunity in Mice With Aging.” Proceedings of the National Academy of Sciences of the United States of America 119, no. 17: e2121028119. 35439062 10.1073/pnas.2121028119 PMC9169949

[acel70000-bib-0117] Sonar, S. A. , M. Watanabe , and J. Nikolich . 2023. “Disorganization of Secondary Lymphoid Organs and Dyscoordination of Chemokine Secretion as Key Contributors to Immune Aging.” Seminars in Immunology 70: 101835. 37651849 10.1016/j.smim.2023.101835 PMC10840697

[acel70000-bib-0118] Stebegg, M. , A. Bignon , D. L. Hill , et al. 2020. “Rejuvenating Conventional Dendritic Cells and T Follicular Helper Cell Formation After Vaccination.” eLife 9: e52473. 32204792 10.7554/eLife.52473 PMC7093110

[acel70000-bib-0119] Takasugi, M. 2018. “Emerging Roles of Extracellular Vesicles in Cellular Senescence and Aging.” Aging Cell 17, no. 2: e12734. 29392820 10.1111/acel.12734 PMC5847882

[acel70000-bib-0120] Takeda, A. , M. Hollmén , D. Dermadi , et al. 2019. “Single‐Cell Survey of Human Lymphatics Unveils Marked Endothelial Cell Heterogeneity and Mechanisms of Homing for Neutrophils.” Immunity 51, no. 3: 561–572.e5. 31402260 10.1016/j.immuni.2019.06.027

[acel70000-bib-0121] Takeuchi, A. , M. Ozawa , G. Cui , K. Ikuta , and T. Katakai . 2021. “Lymph Node Stromal Cells: Diverse Meshwork Structures Weave Functionally Subdivided Niches.” Current Topics in Microbiology and Immunology 434: 103–121. 34850284 10.1007/978-3-030-86016-5_5

[acel70000-bib-0122] Takeuchi, A. , M. Ozawa , Y. Kanda , et al. 2018. “A Distinct Subset of Fibroblastic Stromal Cells Constitutes the Cortex‐Medulla Boundary Subcompartment of the Lymph Node.” Frontiers in Immunology 9: 2196. 30333825 10.3389/fimmu.2018.02196 PMC6176096

[acel70000-bib-0123] Tanriover, C. , S. Copur , A. Mutlu , et al. 2023. “Early Aging and Premature Vascular Aging in Chronic Kidney Disease.” Clinical Kidney Journal 16, no. 11: 1751–1765. 37915901 10.1093/ckj/sfad076 PMC10616490

[acel70000-bib-0124] Tewalt, E. , J. Cohen , S. Rouhani , and V. Engelhard . 2012. “Lymphatic Endothelial Cells—Key Players in Regulation of Tolerance and Immunity.” Frontiers in Immunology 3: 305. 23060883 10.3389/fimmu.2012.00305 PMC3460259

[acel70000-bib-0125] Thompson, H. L. , M. J. Smithey , C. D. Surh , and J. Nikolich‐Žugich . 2017. “Functional and Homeostatic Impact of Age‐Related Changes in Lymph Node Stroma.” Frontiers in Immunology 8: 706. 28659930 10.3389/fimmu.2017.00706 PMC5469916

[acel70000-bib-0126] Thompson, H. L. , M. J. Smithey , J. L. Uhrlaub , et al. 2019. “Lymph Nodes as Barriers to T‐Cell Rejuvenation in Aging Mice and Nonhuman Primates.” Aging Cell 18, no. 1: e12865. 30430748 10.1111/acel.12865 PMC6351843

[acel70000-bib-0127] Torrance, B. L. , A. N. Cadar , D. E. Martin , et al. 2023. “Senolytic Treatment With Dasatinib and Quercetin Does Not Improve Overall Influenza Responses in Aged Mice.” Frontiers in Aging 4: 1212750. 10.3389/fragi.2023.1212750. 37396956 PMC10313122

[acel70000-bib-0128] Turner, V. M. , and N. A. Mabbott . 2017. “Structural and Functional Changes to Lymph Nodes in Ageing Mice.” Immunology 151, no. 2: 239–247. 28207940 10.1111/imm.12727 PMC5418465

[acel70000-bib-0129] Wang, X. , B. Cho , K. Suzuki , et al. 2011. “Follicular Dendritic Cells Help Establish Follicle Identity and Promote B Cell Retention in Germinal Centers.” Journal of Experimental Medicine 208, no. 12: 2497–2510. 22042977 10.1084/jem.20111449 PMC3256970

[acel70000-bib-0130] Wu, R. , F. Ma , A. Tosevska , et al. 2020. “Cardiac Fibroblast Proliferation Rates and Collagen Expression Mature Early and Are Unaltered With Advancing Age.” Journal of Clinical Investigation Insight 5, no. 24: e140628. 10.1172/jci.insight.140628. 33180747 PMC7819745

[acel70000-bib-0131] Xiang, M. , R. A. Grosso , A. Takeda , et al. 2020. “A Single‐Cell Transcriptional Roadmap of the Mouse and Human Lymph Node Lymphatic Vasculature.” Frontiers in Cardiovascular Medicine 7: 52. 32426372 10.3389/fcvm.2020.00052 PMC7204639

[acel70000-bib-0132] Yanes, R. E. , C. E. Gustafson , C. M. Weyand , and J. J. Goronzy . 2017. “Lymphocyte Generation and Population Homeostasis Throughout Life.” Seminars in Hematology 54, no. 1: 33–38. 28088985 10.1053/j.seminhematol.2016.10.003 PMC5260809

[acel70000-bib-0133] Yang, C. Y. , T. K. Vogt , S. Favre , et al. 2014. “Trapping of Naive Lymphocytes Triggers Rapid Growth and Remodeling of the Fibroblast Network in Reactive Murine Lymph Nodes.” Proceedings of the National Academy of Sciences of the United States of America 111, no. 1: E109–E118. 24367096 10.1073/pnas.1312585111 PMC3890876

[acel70000-bib-0134] Yang, J. X. , J. C. Tseng , G. Y. Yu , et al. 2022. “Recent Advances in the Development of Toll‐Like Receptor Agonist‐Based Vaccine Adjuvants for Infectious Diseases.” Pharmaceutics 14, no. 2: 423. 35214155 10.3390/pharmaceutics14020423 PMC8878135

[acel70000-bib-0135] Yip, L. , R. J. Creusot , C. T. Pager , P. Sarnow , and C. G. Fathman . 2013. “Reduced DEAF1 Function During Type 1 Diabetes Inhibits Translation in Lymph Node Stromal Cells by Suppressing Eif4g3.” Journal of Molecular Cell Biology 5, no. 2: 99–110. 22923498 10.1093/jmcb/mjs052 PMC3604916

[acel70000-bib-0136] Youm, Y. H. , T. L. Horvath , D. J. Mangelsdorf , S. A. Kliewer , and V. D. Dixit . 2016. “Prolongevity Hormone FGF21 Protects Against Immune Senescence by Delaying Age‐Related Thymic Involution.” Proceedings of the National Academy of Sciences of the United States of America 113, no. 4: 1026–1031. 26755598 10.1073/pnas.1514511113 PMC4743827

[acel70000-bib-0137] Yu, Y. , and S. Zheng . 2019. “Research Progress on Immune Aging and Its Mechanisms Affecting Geriatric Diseases.” Aging Medicine 2, no. 4: 216–222. 34553108 10.1002/agm2.12089 PMC8445044

[acel70000-bib-0138] Zeng, M. , M. Paiardini , J. C. Engram , et al. 2012. “Critical Role of CD4 T Cells in Maintaining Lymphoid Tissue Structure for Immune Cell Homeostasis and Reconstitution.” Blood 120, no. 9: 1856–1867. 22613799 10.1182/blood-2012-03-418624 PMC3433090

[acel70000-bib-0139] Zeng, M. , A. J. Smith , S. W. Wietgrefe , et al. 2011. “Cumulative Mechanisms of Lymphoid Tissue Fibrosis and T Cell Depletion in HIV‐1 and SIV Infections.” Journal of Clinical Investigation 121, no. 3: 998–1008. 21393864 10.1172/JCI45157 PMC3049394

[acel70000-bib-0140] Zhang, L. , L. E. Pitcher , V. Prahalad , L. J. Niedernhofer , and P. D. Robbins . 2023. “Targeting Cellular Senescence With Senotherapeutics: Senolytics and Senomorphics.” FEBS Journal 290, no. 5: 1362–1383. 35015337 10.1111/febs.16350

[acel70000-bib-0141] Zhang, Y. M. , X. M. Chen , D. Wu , et al. 2005. “Expression of Tissue Inhibitor of Matrix Metalloproteinases‐1 During Aging in Rat Liver.” World Journal of Gastroenterology 11, no. 24: 3696–3700. 15968723 10.3748/wjg.v11.i24.3696 PMC4316019

[acel70000-bib-0142] Zou, M. , C. Wiechers , and J. Huehn . 2021. “Lymph Node Stromal Cell Subsets‐Emerging Specialists for Tailored Tissue‐Specific Immune Responses.” International Journal of Medical Microbiology 311, no. 3: 151492. 33676241 10.1016/j.ijmm.2021.151492

